# Shannon sampling based approach for the structural solution of nano-objects by laboratory and synchrotron SAXS data

**DOI:** 10.1107/S1600576726004358

**Published:** 2026-07-07

**Authors:** Liberato De Caro, Domenico Alberga, Francesco Scattarella, Teresa Sibillano, Vincenzo Mangini, Boyang Zhou, Thibaud Stoll, Cinzia Giannini

**Affiliations:** a Institute of Crystallography National Research Council Bari 70126 Italy; b Excelsus Structural Solutions (Swiss) AG, Park Innovaare, Villigen, 5234, Switzerland; ESRF, France

**Keywords:** SAXS, small-angle X-ray scattering, PS20 micelles, VitE-TPGS micelles, Nyquist–Shannon sampling, proteins

## Abstract

We propose a Shannon sampling based approach for the analysis of laboratory and synchrotron small-angle X-ray scattering (SAXS) data. This method avoids calculation of the pair distance distribution function *P*(*r*), which is typically used as an alternative to the model-dependent fitting of SAXS data for determining the size and shape of the scattering object. Using this new approach it is also possible to skip any model-dependent fit of the SAXS data. The method has been tested on SAXS data collected from Polysorbate 20, d-α-tocopheryl polyethyl­ene glycol 1000 succinate spheroidal core–shell micelles and relevant well known proteins (lysozyme, apoferritin, carbonic anhydrase 2) of different molecular weights. The results confirm the potential of the approach, even for laboratory scattering data and in the presence of polydispersity. The method allows the reliable derivation of maximum size, mass, volume, polar/equatorial asymmetry, hydration fraction, shell thickness and core/shell electron-density differences for core–shell structures, directly in the *q* domain, working on SAXS data measured from dilute samples for which interparticle structure factors are negligible.

## Abstract 2

We present a model-independent approach based on the Shannon sampling theorem to retrieve key structural parameters of proteins and core–shell micelles directly from small-angle X-ray scattering (SAXS) profiles. By bypassing the pair distribution function, the method overcomes *q*-truncation artifacts and provides reliable mass, size and aggregation number estimates even from noisy laboratory data. This formalism enables fast, robust and high-throughput analysis for both *in situ* and *operando* experiments.

## 1. Introduction

Small-angle X-ray scattering (SAXS) is a non-destructive, indispensable tool for quantitative nanoscale characterization of soft matter and complex fluids, including micelles, colloids and macromolecules. By meticulously controlling sample preparation – typically using thin-walled capillaries to minimize background and ensure optimal transmission – and employing radiation-controlled exposure strategies, SAXS yields robust structural/morphological information on the scattering nano-objects under investigation, dispersed in a buffer. Data can be collected either on a relative scale, *i.e.*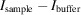, or on an absolute scale, as 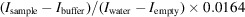
 cm^−1^, where the normalization to water scattering is based on the isothermal compressibility of water. The value 0.0164 cm^−1^ corresponds to the theoretical absolute scattering intensity of pure water at 298 K and atmospheric pressure (Orthaber *et al.*, 2000 ▸; Zeng *et al.*, 2017 ▸; Roe, 2000 ▸).

Data interpretation in SAXS relies on the form factor, which describes the scattering from an individual particle, combined with the structure factor – when interparticle interactions are present – to account for those interactions. When interparticle effects are minimized for sufficiently dilute sample solutions, the scattering curve primarily reflects the form factor and, therefore, the shape of the particle. For the form factor, analytical expressions are available to compute the scattering intensity and describe the object shape (sphere, disc, rod, dumbbell, core–shell *etc*.), often incorporating polydispersity parameters such as size, shape and shell thickness.

The form factor modeling approach has successfully allowed the morphological characterization of spheroidal core–shell micelles, enabling the determination of micellar core size, shell thickness and electron-density contrast. This required the definition of *a priori* assumptions concerning particle shape, polydispersity, uniformity, layer sharpness and interparticle interactions (Pedersen, 1997 ▸; Glatter & Kratky, 1982 ▸; Castelletto & Hamley, 2002 ▸). In practice, some of these *a priori* assumptions may not hold, which can invalidate the determination of key structural parameters of unknown scattering nano-objects from SAXS data.

Alternatively, a second class of methods can be used, which builds on the pair distance distribution function 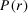 computed through indirect Fourier transform of the SAXS data. These techniques avoid shape assumptions but still yield insights into the maximum particle size 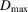, radius of gyration 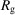 and internal inhomogeneities (Svergun, 1992 ▸; Hansen, 2000 ▸; Vestergaard & Hansen, 2006 ▸). Within this second class, a theoretical calculation of 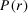, compared with the evaluation of experimental 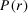 moments, was recently proposed as a possible alternative (De Caro *et al.*, 2025 ▸).

More advanced frameworks employ hybrid or simulation-based models, combining SAXS data with molecular dynamics or Monte Carlo simulations. These methods generate structural ensembles consistent with scattering patterns and capture effects such as dynamic fluctuations, anisotropy in the shell region, or non-ideal solvation (Stuhrmann, 2008 ▸; Chen *et al.*, 2023 ▸; Cezar *et al.*, 2025 ▸). Moreover, contrast variation and global fitting approaches integrate SAXS with complementary scattering techniques (*e.g.* small-angle neutron scattering, SANS) or with multiple experimental datasets at once, thereby improving robustness and reducing the degeneracy of solutions (König *et al.*, 2020 ▸). In fact, in SAXS model fitting, unconstrained least-squares minimization often yields degenerate solutions. To stabilize and obtain physically meaningful results, one needs to add constraints to the χ^2^ minimization (Svergun, 1992 ▸; Pedersen, 1997 ▸; Stuhrmann, 2008 ▸; Breßler *et al.*, 2015 ▸; König *et al.*, 2020 ▸).

In the present work, we propose a new method based on the Nyquist–Shannon (NS) sampling theorem. The new approach works directly on the SAXS data, as collected in the *q* domain, without any need for calculating distributions of distances in real space, namely the pair distance distribution function 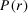. The method proved to be effective for deriving many key parameters from SAXS data measured on dilute samples, for which interparticle structure factors are negligible: maximum size of the scattering particle, 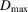, polar/equatorial asymmetry (ellipticity), volume, mass and number of monomers when needed. We successfully tested this new approach on Polysorbate 20 (PS20) and vitamin E d-α-tocopheryl polyethyl­ene glycol succinate (VitE-TPGS) micelles, using both laboratory and synchrotron SAXS data, and on relevant well known proteins (lysozyme, apoferritin, carbonic anhydrase 2) from synchrotron SAXS data. Moreover, we have checked the reliability of the structural parameters derived by the new approach by also determining them by means of a model-dependent least-squares χ^2^ minimization of the difference profile between experimental and calculated SAXS data. Using the new proposed approach, it is straightforward to derive several key structural parameters of unknown scattering nano-objects from SAXS data; it could also be thought of as a preliminary and quick way to obtain a first estimate of these parameters, before performing time-consuming least-squares χ^2^ minimization of the difference profile between experimental and calculated SAXS data. Additionally, it could be considered a useful independent and indirect check of the *a priori* assumptions concerning particle shape, polydispersity, uniformity, layer sharpness *etc*., which usually characterize SAXS modeling approaches.

## 2. Theoretical framework

In the following sections we will present the new approach, based on the NS sampling of SAXS data, reporting the main formulae and leaving further mathematical details to the supporting information.

### 2.1. Determination of the maximum size of the scattering particle in *q* space

The aim of this section is to demonstrate that it is possible to obtain a reliable estimate of 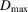, the maximum size of the scattering particle from SAXS data, without calculating 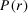, through a new approach based on the NS sampling of SAXS intensities.

Moore (1980 ▸) used the Shannon sampling theorem (Shannon, 1948 ▸) to sample the SAXS profile at a selection of *q* values, called the Shannon channels, here denoted as NS channels (Moore, 1980 ▸; Grant, 2022 ▸), defined by
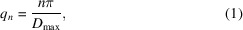
with *n* a positive integer and 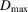 the maximum particle dimension.

The SAXS profile 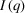 can be described as an infinite sum of the intensities sampled at 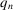, *i.e.*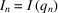 (Grant, 2022 ▸):
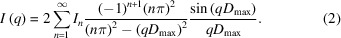
Application of the NS sampling to SAXS data has been shown to be advantageous because it reduces a densely measured and noisy scattering curve to a small set of independent points that fully capture the real structural information, avoiding risks of overfitting. In fact, the structural information content in the SAXS profiles is affected by rotational averaging and other physical factors, thus giving experimental access to only a limited number of NS channels (up to 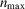). However, the number of experimentally available NS channels, 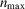, is much lower than the typical number of measured intensities (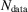) in a SAXS experiment. A measurement step Δ*q* ∼ 0.005 Å^−1^ of 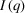 yields an oversampling ratio OR = 
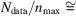

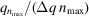
 of the information (signal) contained in the experimental SAXS intensity. This redundancy is crucial for robustly constraining the determination of NS channel intensities 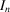, even in the presence of low signal-to-noise ratios. With typical oversampling factors of 50−100, the information content within the SAXS profile is significantly redundant; this compensates for the low signal-to-noise ratio inherent in the determination of the NS channel intensities, in particular for noisy laboratory measurements.

Moreover, considering that the scattering intensity 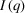 decreases rapidly with increasing magnitude of the scattering vector *q* (typically following a high negative power of *q*), often also very few NS channels are sufficient to calculate reliably average quantities determined by the SAXS intensity. In fact, the pair distance distribution function 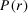, calculated as a Fourier transform of a truncated version of the infinite series in equation (2), containing only a finite number of terms 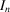, *i.e.* those experimentally available, suffers very often from severe ripples caused by Fourier termination effects. These ripples significantly affect the 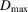 determination, *i.e.* where 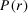 goes to zero (Grant, 2022 ▸).

Nevertheless, the NS sampling approach offers the possibility to determine the forward scattering intensity 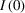, the average vector length 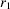 in the nanoparticle and the gyration ratio 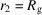, evaluated as sums over the NS channel intensities (Grant, 2022 ▸), up to the 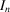 experimentally available. These can be defined by
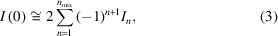

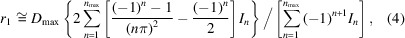

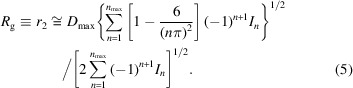
These quantities are, respectively, moments of order 0, 1 and 2 of 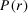. The 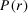 moments are mean values, related to integrals of the function 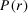 multiplied by some power of *r*. Being integral quantities, they are much less affected by truncation effects due to the finite 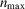 experimentally available.

Equations (3)–(5) allow structural quantities to be derived directly from the SAXS profiles measured on dilute samples, for which interparticle structure factors are negligible, if 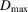 has been previously estimated. The Guinier plot (Guinier & Fournet, 1955 ▸) permits evaluation of 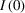 and *R*_g_ directly from the experimental SAXS profile at very low *q* (in the asymptotic limit *q* →0). These values are related to a low-spatial-resolution approximation of the shape of the scattering particle, therefore requiring a small number of terms in the sum expressions of equations (3)–(5).

Thus, in that case, starting from equations (3) and (5), we can write the following two equations:
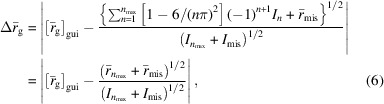

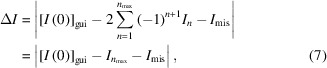
where 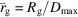 is the normalized gyration radius, and
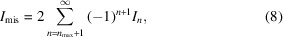

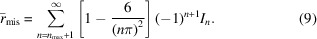
Here, ‘mis’ refers to ‘missing’ intensities, *i.e.* related to SAXS intensity values which are unobserved due to the noise affecting measurements, and the subscript gui refers to the Guinier plot. However, for 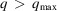 the SAXS intensities are orders of magnitude smaller than the measured values for 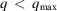. For scattering nano-objects we usually have a 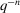 scaling of intensities, as a function of *q*, with *n* larger than 1, up to 4 for ideally well defined surfaces of globular objects (Porod law). Moreover, there are ‘alternating signs’ in the numerical series. Therefore, 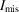 and 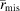 are expected to be very small.

For this reason, they can also be estimated assuming a power-law *q* scaling of the unobserved intensities:
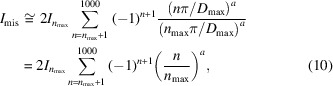

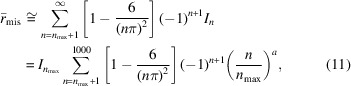
with 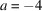, or different suitable powers that could be chosen in the case of additional information about the shape or other structural properties of the scattering nano-objects, or after having calculated the Kratky plot (Glatter & Kratky, 1982 ▸). The upper limit in the sums can be chosen arbitrarily. The value of 1000 in the above equations is equivalent to having an infinite upper limit in the sums. Our experimental tests have demonstrated that the small contributions 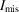 and 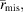 respectively, in equations (6) and (7), can also be very often neglected, *i.e.* the truncation at 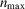 of equations (6) and (7) already gives reliable results of the 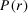 moments (see Table 1 ▸).

By varying 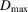, *e.g.* in small steps of 0.1 Å, a value usually smaller than the experimental error with which 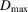 can be determined, we obtain from equations (6) and (7) two dimensionless functions, 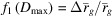 and 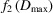 = 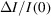, whose minima give two possible values, 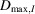 and 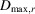, of the unknown maximum size. We can search for the two minima by changing 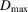 in the range from 2.5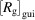 to 3.5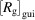, to obtain 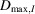 and 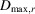 which minimize equation (6) and equation (7), respectively, or in other suitable intervals, if needed, when the minima fall out of the above interval. To find these minima, smoothed intensity values are used to reduce the effect of noise, an operation allowed due to the large oversampling of the SAXS signal. For this purpose, *q*-adaptive filtering sigmoidal kernels, described in the supporting information (Section S2), can be used. This process leaves lower-*q* SAXS intensities unchanged, averaging only the noisy region of the experimental data at higher *q*. Utilizing mean-filtered SAXS profiles ensures statistically robust evaluations of NS channel intensities. The large oversampling allows for local averaging around each 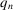 point, thereby incorporating neighboring intensities and enabling the extraction of reliable 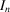 values from the noisy data.

The experimental profile is measured on a discrete set of scattering vector magnitudes 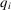, with *i* ∈ (1, *i*_max_). For any considered 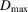, the smoothed SAXS profile will usually not contain an intensity value *I*(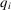) located exactly at the 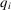 value of the *n*th NS channel, *i.e.* corresponding to 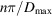. We can therefore use the intensity value from the smoothed SAXS profile whose 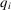 is closest to 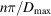, *i.e.* the one minimizing 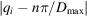. The step size of 0.1 Å may be too fine for identifying the minima of the functions 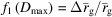 and 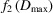 = 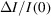. Therefore, both functions also usually require smoothing, *e.g.* with a mean filter. Different levels of smoothing of 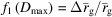 and 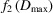 = 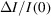 may yield slightly different solutions, 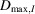 and 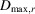. Among all the solutions obtained by varying the width of the mean filter, we can select only the values of 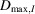 and 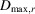 that satisfy the following condition:
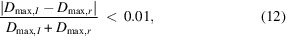
*i.e.* the values of 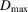 that minimize simultaneously both 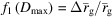 and 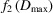 = 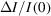.

**Figure 1:**
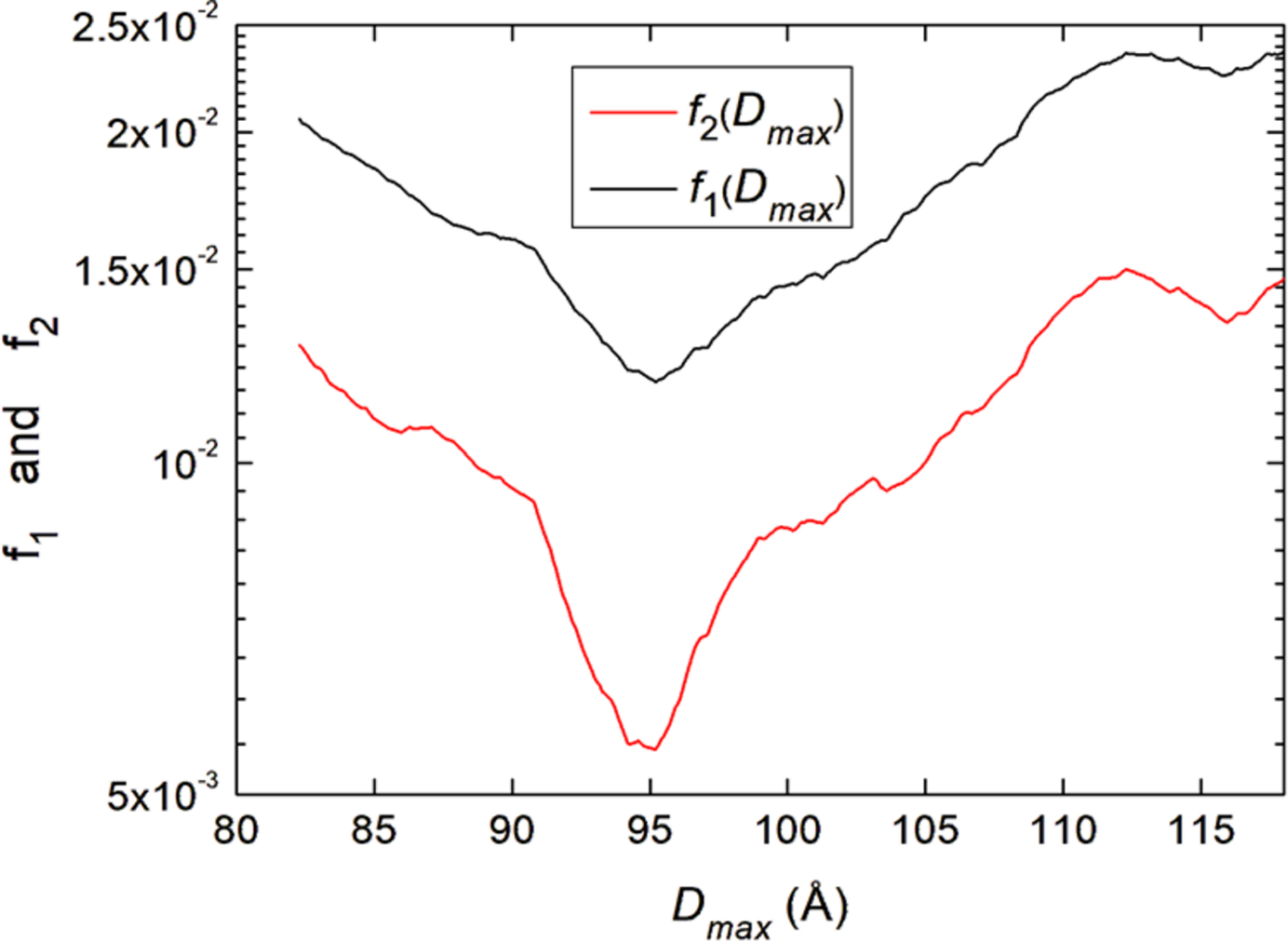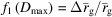 (black curve) and 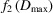 = 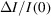 (red curve) as a function of 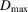 for laboratory SAXS data of PS20 micelles. The minima of the two functions give the maximum size of the PS20 micelles.

As an example, Fig. 1 ▸ shows the functions 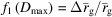 (black curve) and 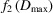 = 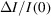 (red curve) evaluated for laboratory SAXS data collected from PS20 micelles. The experimental conditions used for measuring the SAXS data are described in Section 3.

By changing the level of smoothing of the two functions, we could find several solutions satisfying both equations (6) and (7) and the constraint given by equation (12). The average values 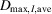 and 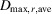 of all minima of equations (6) and (7), satisfying also the condition given by equation (12), will give the maximum size of the nano-objects under study, without the need to calculate the pair distance distribution function:
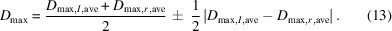
When the Guinier plot is not accurately evaluated, *e.g.* for inaccurate experimental measurements at low *q*, due to incorrect sample preparation or experimental settings, we have no solution satisfying equation (12), because the two minima are not aligned in terms of abscissa coordinates, *i.e.*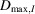 is too different from 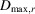. Therefore, the result of having the two minima perfectly aligned, as shown in the example of Fig. 1 ▸, is an indirect confirmation that the Guinier plot has been correctly computed, *i.e.* sample and experiment preparation have been correctly performed. When this condition is not satisfied, the Guinier plot should be recalculated, usually eliminating some SAXS intensities at very low *q*, leading to solutions satisfying the constraint given by equation (12), or alternatively repeating sample preparation or data collection. This finding provides an internal check that 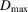, 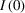 and 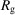 have been consistently determined with respect to each other. Every time the SAXS data allow a meaningful Guinier plot to be obtained, giving 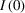 and 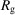 values truly representative of the scattering nano-objects, the approach described above gives the possibility to determine a 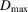 value consistent with these values.

Table 1 ▸ shows the results obtained for 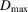 for several proteins and core–shell micelles, using laboratory and synchrotron SAXS data. For comparison, we report also the gyration radius and the *I*(0) value determined by the described approach based on the NS sampling theorem, as well as the values reported in the Small Angle Scattering Biological Data Bank (SASBDB) repository (https://www.sasbdb.org/; Valentini *et al.*, 2015 ▸), obtained by other software (*GNOM*) (Svergun, 1992 ▸). Although different approaches give different 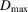, the differences are within the experimental error bars. Thus, we can conclude that there is a very good agreement.

Moreover, the estimation of the truncation errors due to the finite maximum *q* value experimentally available, reported in Table 1 ▸, shows that they usually are less than 1%. In any case, we have used these small corrections only for the determination of 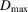. Conversely, the values of *I*(0) and *R*_g_ reported in Table 1 ▸ have been obtained without considering these small corrections due to finite 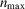. Even without these corrections, the results shown in Table 1 ▸ demonstrate that the predicted values for these quantities obtained by the NS sampling formulae agree with the values obtained by the Guinier plot, within the experimental errors. These results show that truncation of the series does not significantly affect the *P*(*r*) moments *I*(0) and 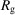, as these quantities are related to integrals of *P*(*r*).

### 2.2. Determination of the mass and polar/equatorial asymmetry of core–shell structures

The aim of this section is to demonstrate that it is possible to obtain relevant structural information on core–shell structures (mass, volume, ellipticity) through the new approach, based on the NS sampling of SAXS data.

The volume *V* of a protein of a nearly globular shape, proportional to the ratio between the scattering intensity at *q* = 0, *I*(*0*), and the Porod invariant *Q =*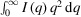, can, in principle, be determined directly from SAXS data, even collected in relative units (Fischer *et al.*, 2010 ▸):
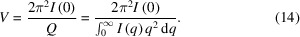
The protein molecular weight can be obtained from the volume *V* (Å^3^), given by equation (14), multiplied by the average density 0.83 × 10^−3^ kDa Å^−3^ for proteins in solution, equivalent to a mass density ρ_p_ = 1.37 g cm^−3^ (Fischer *et al.*, 2010 ▸). The calculation of the Porod invariant *Q* can be performed up to the 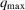 accessible in the SAXS data, giving an apparent protein volume 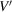, instead of the correct volume *V*, under the hypothesis of a protein globular shape. However, a linear relationship has been derived by Fischer *et al.* (2010 ▸), to obtain the correct volume from the apparent value 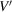:
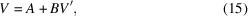
where *V*, *A* and 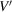 are expressed in Å^3^, and *B* is dimensionless. The coefficients of the linear regression, *A* and *B*, depend on the maximum scattering vector magnitude 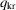 used for the calculation of the invariant *Q* (*i.e.* the integral of the Kratky plot) (Fischer *et al.*, 2010 ▸). We note that 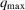 corresponding to the maximum NS channel available in the SAXS data is usually different from 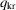, used for the calculation of the Porod invariant *Q*. 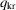 can be estimated by comparing 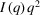 at any *q* and the mean value 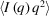, as shown, for example, in the supporting information for apoferritin (SASDFN8) (see Section S4). The scattering vector value where 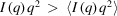 allows one to estimate 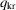, *i.e.* where the level of noise prevents access to further structural information from the 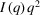 profile.

Applying the NS formalism, the following expressions for 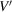, *V* and the molecular weight *M*_p_ can be derived (Grant, 2022 ▸):
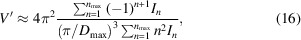

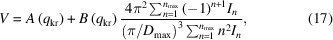

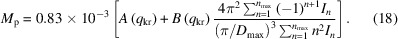
Here we apply a linear fit of *A* and *B* values reported by Fischer *et al.* (2010 ▸), where 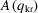 has the dimension of a volume (Å^3^) and 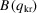 is dimensionless. The linear fit gives
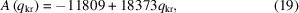

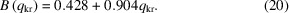
Corrections like 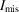, related to the finite 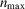 of the experimental SAXS data, are not needed in equations (17)–(18), because the corrections due to the finite *q* range are already considered through the correction 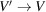, by means of equations (18)–(20).

The determination of the maximum size, 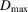, and of the Shannon channel intensities obtained with the approach described in the previous section allows us to determine the molecular weight for biological macromolecules and proteins.

For estimating the molecular weight of macromolecules another invariant can be introduced, named the correlation volume 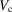 but with a surface (length^2^) unit, 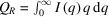 (Rambo & Tainer, 2013 ▸), which is useful when the *Q* invariant diverges at high scattering vectors in the Kratky plot. The use of this invariant leads to the following mass formula, which is concentration independent, holds also for SAXS data in relative units and gives the nano-object volume (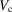) per self-correlation length (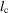) (Rambo & Tainer, 2013 ▸):
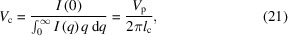

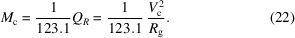
Here, 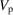 is the effective protein volume, which could be different from *V* obtained by the Porod invariant [equation (17)], due to its flexibility. Equation (22) has been derived by Rambo & Tainer (2013 ▸) through a comparison of the new invariant 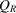 with the mass of many proteins taken from the Protein Data Bank (https://www.rcsb.org/). 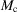 as obtained by equation (22) is given in kDa if 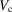 is expressed in Å^2^ and 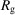 in Å. For mixed protein–nucleic acid complexes or RNA alone, other formulae can be derived, with different fitting power-law relationships (Rambo & Tainer, 2013 ▸).

The NS formalism also allows us to express the above quantities in terms of sums on the NS channels (Grant, 2022 ▸):
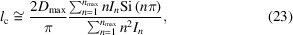

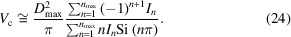

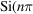
) is the sine integral function calculated at 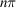. In equations (23) and (24) we have approximated the infinite sum up to 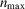, because equation (22) already takes into account the effects of the finite maximum value of *q*, since it has been obtained by the fit over thousands of macromolecules always characterized by a finite 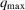 (Rambo & Tainer, 2013 ▸).

For spherical macromolecules we have 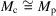. For spheroidal shapes with sufficiently large polar/equatorial asymmetry and for flexible macromolecules, we have 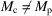. In principle, given 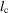 and 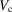, 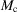 could give indications about the internal spatial correlation, *i.e.* the effective extension and the coherence of the scattering of any nanoparticle. Instead, equation (17) gives the effective volume, corrected by the effect of a finite 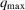. This effective volume is that of an equivalent sphere with the same scattering as the nanoparticle. Furthermore, given *V*, if the particle is not a protein, we could obtain its mass from equation (18) by inserting the correct value of the particle-mass density, substituting 1.37 g cm^−3^ for the mass density 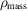 of the molecules constituting the particle, *i.e.* generalizing equation (18) as follows:
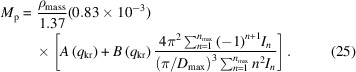
These considerations allow us to extend the range of application of the above formulae to predict the mass of core–shell spheroidal micelles. In the supporting information we have shown how to derive the polar/equatorial asymmetry (ellipticity) directly by the comparison of 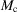 and 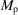. The ratio of 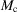 and 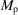 for spheroidal nano-objects, where 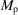 can be determined from equation (25), can be related just to the polar/equatorial asymmetry of its shape. When applied to core–shell structures [equation (S37) in Section S5 of the supporting information], in the case of prolate shapes, this ratio gives simply the particle ellipticity; for oblate shapes this ratio gives 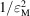:
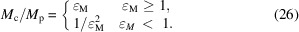


We have tested this approach on:

(i) Well known proteins (lysozyme, apoferritin, carbonic anhydrase 2) of different molecular weights, whose synchrotron SAXS data were downloaded from the SASBDB repository.

(ii) The core–shell nano-objects studied in this work, *i.e.* apoferritin (SASDFN8), PS20 and VitE-TPGS micelles (laboratory and synchrotron SAXS data).

Results are summarized in Table 2 ▸. We observe a good agreement between 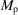 and *M* (experimental mass value reported in the SASBDB repository) for the proteins, and between 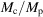 and 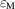 for core–shell structures (apoferritin and micelles only), with the values derived from a model-dependent fit of the experimental SAXS data, described in detail in the following.

Conversely, for the proteins that do not have a core–shell structure, the ratio of the two masses is an indication of the actual internal scattering correlation compared with the value corresponding to an ideal compact scattering object, assumed for the calculation of 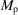.

### 2.3. Determination of the volume, hydration fraction and aggregation number of core–shell structures

The aim of this section is to demonstrate that it is possible to determine the number of monomers or sub-units constituting the core–shell structure through the new approach based on the Shannon sampling formalism of SAXS data, combined with the volume of the scattering particle and its hydration fraction. All these quantities can be derived without the need to perform model-dependent simulations.

The knowledge of the polar/equatorial asymmetry, obtained by equation (26) (penultimate column of Table 2 ▸), and of the maximum size (third column of Table 1 ▸) for spheroidal core–shell structures allows the calculation of its volume. Moreover, Rambo & Tainer (2013 ▸) have shown that, for mixed protein–nucleic acid complexes or single RNA molecules, other formulae than equation (22) can be derived, with different fitting power-law relationships of 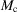. We could verify if, for core–shell structures, there is a power-law relation of 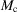 suitable to derive the non-hydrated mass of the nanostructure and, consequently, the number of monomers constituting the core–shell structures. We would expect that the internal scattering coherence (related to 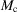) should be quantitatively related to the number of monomers 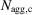 packed in the structure, because in spheroidal micelles the monomers do not pack necessarily with an isotropic order. We have already shown that for core–shell nanostructures the ratio 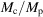 is related to the ellipticity [equation (26)]. Therefore, the non-hydrated mass 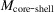 of core–shell structures and, consequently, the number of monomers 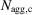 could be derived from the following equation:
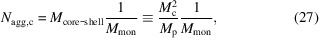
where 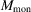 is the molar weight mass of the monomer constituting the core–shell structure, and 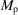 is given by equation (25). The above formula is related to quantities 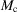 and 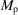, which can be derived also by SAXS data on a relative scale. Therefore, equation (27) could be useful to estimate the aggregation number from SAXS data even collected on a relative scale.

For SAXS intensity on an absolute scale an independent evaluation of the aggregation number can be determined as
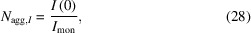
where 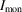 is the *q*→0 value of the SAXS intensity of one monomer and can be estimated as follows (De Caro *et al.*, 2024 ▸):
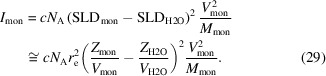
*c* is the concentration of monomers (g cm^−3^), 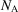 the Avogadro number, 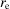 the classical electron radius, SLD the scattering length density for the monomer and the water molecule (9.44 × 10^10^ cm^−2^), 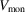 the monomer volume, 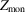 the total number of electrons in a monomer, 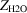 the total number of electrons in a water molecule, and 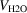 the volume of a water molecule. The comparison between the aggregation numbers obtained by the two independent formulae, *i.e.* equation (27) versus equations (28)–(29), is useful for validating the prediction from the new formula [equation (27)].

Moreover, we note that the aggregation numbers derived from the above formulae are independent of the particle volume. By using equation (27), the hydrated fraction of the core–shell volume can be calculated from the ratio between the volume of monomers and the volume 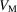 of the core–shell structure, where 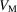 can be readily derived from 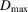 and ɛ_M_ [equation (26)]:
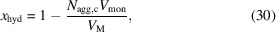

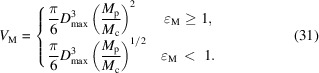
The results obtained by the above formulae are summarized in Table 3 ▸.

Note that we obtain hydrated volume fractions for the micelles that are too high for the oblate shape compared with the prolate values. This finding implies that the prolate shape is the correct one. For the apoferritin, we obtain a spherical shape, as expected. The new method proposed here for determining prolate/oblate shapes of spheroidal micelles can be considered an alternative approach to existing ones (*e.g.* Vass *et al.*, 2008 ▸).

Furthermore, the results of Table 3 ▸ show that the estimation of the aggregation number 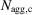 obtained from the new formula proposed here [equation (27)] seems to give reliable numbers of monomers constituting the core–shell nano­struc­tures, independently of those estimated through equations (28)–(29). Within the errors, equation (27) gives the correct number of sub-units, *N* = 24, constituting apoferritin.

Moreover, the comparison with *N*_agg_ = 127 ± 2 obtained for a sample of VitE-TPGS of the same concentration and the same 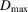, but derived by a different approach based on the *P*(*r*) moments (De Caro *et al.*, 2025 ▸), is an important confirmation of the new estimations obtained by equation (27) and equations (28)–(29) through the NS sampling approach proposed here. Also, the comparison of *N*_agg_ = 45 for PS20 with the results obtained in other work (De Caro *et al.*, 2024 ▸; Ehrit *et al.*, 2023 ▸) confirms the same conclusion about the reliability of the *N*_agg_ estimations here proposed. The 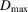 of the PS20 micelles characterized here is about 1 nm larger than that of the PS20 micelles studied by De Caro *et al.* (2024 ▸) and Ehrit *et al.* (2023 ▸), implying a volume about 33% greater and, consequently, 33% more monomers constituting the micelles. The value of *N*_agg_ = 34 ± 1 reported by De Caro *et al.* (2024 ▸) and Ehrit *et al.* (2023 ▸), incremented by one-third, gives *N*_agg_ ∼ 45, *i.e.* the same as the value obtained here, but by completely different approaches. All these findings confirm the validity of the new proposed formula for estimating the aggregation number of core–shell nanostructures from SAXS data collected on a relative scale [equation (27)]. Future use of this formula will help to expand and further validate its applicability.

### 2.4. Determination of the shell thickness of core–shell structures

The aim of this section is to demonstrate that it is possible to determine the shell thickness of core–shell structures, by solving either numerically or graphically an equation based on the balance of the volume of the monomers and the volume of the hydrated molecules inside the core–shell structures.

**Figure 2:**
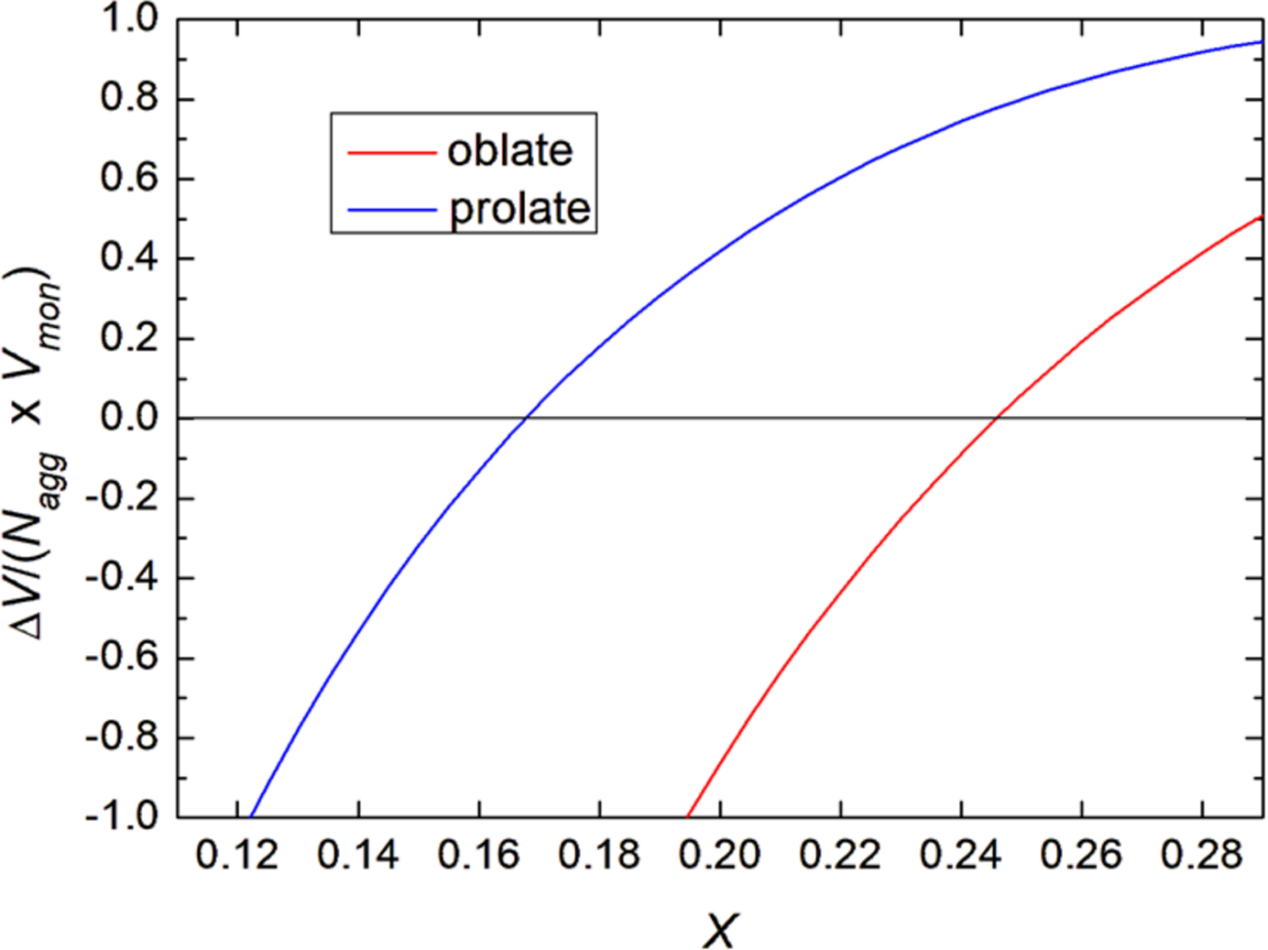
Δ*V*/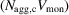 as a function of *X*, for the PS20 laboratory data, for the prolate (blue) and oblate (red) shapes. The solution of Δ*V* = 0 for the prolate shape is obtained for *X* = 0.165 ± 0.005. The solution of Δ*V* = 0 for the oblate shape is obtained for *X* = 0.245 ± 0.005, a non-physical solution leading to a too large shell thickness (about a quarter of the maximum size).

In the supporting information we have derived the following equation, which gives the possibility to derive the fraction 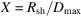 [equation (S10) of the supporting information]:
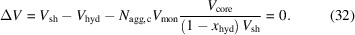


The numerical solution of equation (32) enables the determination of the shell thickness 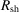 since both the shell and core volumes are a function of *X* [equations (S24)–(S25) and (S27) of the supporting information] and 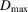 has already been determined. Fig. 2 ▸ shows 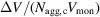 as a function of *X*, for the PS20 laboratory data, for the prolate (blue) and oblate (red) shapes. The results for the considered core–shell structures, including apoferritin, have been summarized in Table 4 ▸.

The prolate solutions for the micelles shown in Table 4 ▸ are in good agreement with the results obtained by the model-dependent simulations, reported in the supporting information (Section S4).

Also, the shell size of the apoferritin, in the core–shell approximation, is correctly derived. For oblate shapes we always obtain values for shell thickness/maximum distance ratios that are too large: for the PS20 laboratory SAXS data *X* = 0.245 ± 0.005; for the PS20 synchrotron SAXS data *X* = 0.245 ± 0.005; for the VitE-TPGS laboratory SAXS data *X* = 0.205 ± 0.005. In all cases, the oblate solution can be discarded because it leads to shell thickness values that are too large, a check that can be readily verified if one has some additional information about the monomers constituting the micelles (*e.g.* estimated hydro­phobic chain length *etc*.).

### 2.5. Determination of shell and core electron-density differences

The aim of this section is to demonstrate that, when SAXS data are available in absolute units, it is possible to determine the core and shell electron-density differences of core–shell structures, by solving either numerically or graphically a suitable equation based on the balance of the total number of electrons inside the volume of the scattering particles.

For a core–shell system, we have derived an equation [see equations (S46)–(S48) of Section S6 of the supporting information] that can be solved numerically to determine the ratio of the two electron-density differences 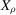
 = 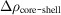/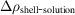.

This equation has been rewritten here in terms of 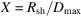:
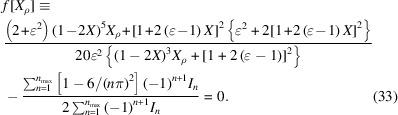


The ratio 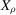 is the solution of the above equation, once 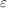 and *X* have been determined, as previously described. 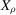 can also be obtained for SAXS data on a relative scale. Conversely, for determining the shell and core electron-density differences, we need SAXS data on an absolute scale. For this purpose, we can use the following equations [equations (S48)–(S49) of Section S6 of the supporting information]:
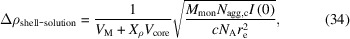

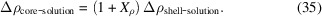
*c* is the monomer concentration (g cm^−3^), 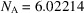
 × 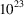
 mol^−1^ is the Avogadro number, 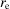 is the classical electron radius (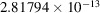
 cm e^−1^), 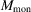 is the molar mass of one monomer and 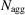 is the number of aggregated monomers within the core–shell structure; 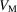 is the volume of the core–shell structure and 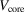 is the core volume.

Table 5 ▸ reports the electron-density differences obtained for the core–shell structures under study. We have a good agreement between 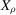 derived here and the values determined by the model-dependent fits of SAXS data, reported in Section S4 of the supporting information, although we note that the solutions of equation (33) are systematically about 5% higher than the values obtained by the fits of SAXS data. This difference could be caused by the presence of polydispersity, as discussed in Section S4.

Nevertheless, we obtain a perfect agreement between the electron-density differences derived here for the VitE-TPGS micelles and those determined by De Caro *et al.* (2025 ▸) for micelles of the same size (Δρ_shell-solution_ = 0.032 ± 0.001 e Å^−3^, Δρ_core-solution_ = −0.026 ± 14;0.001 e Å^−3^). Conversely, for the PS20 micelles, if we compare the results derived here with those published elsewhere (De Caro *et al.*, 2024 ▸; Ehrit *et al.*, 2023 ▸), we note a perfect agreement for the values. Indeed, in their work we find Δρ_core-solution_ = −0.030 ± 0.002 e Å^−3^. Conversely, the value determined by De Caro *et al.* (2024 ▸) of Δρ_shell-solution_ = 0.050 ± 0.005 e Å^−3^ is quite different from the values determined here from both laboratory and synchrotron SAXS data. However, we have already stressed that the PS20 micelles characterized here are about one-third bigger than those characterized by De Caro *et al.* (2024 ▸) and Ehrit *et al.* (2023 ▸). Very recently, Evers *et al.* (2025 ▸) have shown the great variability of the polyoxyethyl­ene (POE) chain with respect to the nominal 20 units, for commercial PS20. For the monolaurate core we should expect a more stable chemical composition and, consequently, a stable value of the core electron-density difference, as obtained by our analysis. The variability of the length and the structure of the POE chains in commercial PS20 products (Evers *et al.*, 2025 ▸) could easily cause different maximum sizes, shell thickness and shell electron density, as verified in the present analysis.

Finally, the application of our approach to apoferritin leads to a very low core electron-density difference, as expected, for the iron cage function of this protein. From the FASTA sequence of the SASDFN8 apoferritin the nominal number of electrons inside one of the 24 units constituting this protein can be estimated as about 10 × 10^3^. This value can be compared with the value 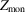 obtained by the electron-density differences derived here, by means of the formula
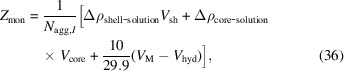
where the last term gives the number of electrons of an equivalent number of water molecules contained in the un-hydrated volume of the protein. By inserting the electron-density differences determined here (Table 5 ▸) and the other quantities reported in the previous tables for apoferritin, we obtain 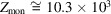.

## 3. Experimental section

In this section we summarize the main experimental data of the SAXS experiments and the results of model-dependent fits of the SAXS experimental data. Further details are reported in the supporting information. The model-dependent fits described here have been performed as a further check of the reliability of the results discussed in Section 2, which are completely independent from the results reported in the following.

### 3.1. Laboratory SAXS data

SAXS data were collected by means of a XEUSS3.0 HR system equipped with a motorized dual-X-ray source (Excillum Ga metal jet and Cu Genix3D micro-sources). All measurements reported here were carried out using Ga *K* radiation (λ = 1.34 Å). Scattering was recorded with an Eiger 2R 1M detector under standard vacuum conditions.

SAXS measurements were performed using 2 mm Biocube capillaries. For PS20 (5 mg ml^−1^), measurements were conducted at a sample-to-detector distance of 600 mm using C1 collimation (1.3 × 1.3 mm beam) with an exposure time of 60 s per frame for 60 frames (1 h total). Additional measurements were performed at 1800 mm using C4 collimation (0.5 × 0.5 mm beam) with an exposure time of 600 s per frame for 60 frames (10 h total).

For VitE-TPGS (0.4 wt%), measurements at 600 mm with C1 collimation (1.3 × 1.3 mm beam) were performed for 60 s per frame for 60 frames (1 h total). Measurements at 1800 mm with C4 collimation (0.5 × 0.5 mm beam) were performed for 300 s per frame for 60 frames (5 h total).

### 3.2. Synchrotron SAXS data

SAXS data were collected at the P12 BioSAXS beamline of the PETRA III synchrotron on the DESY campus in Hamburg, Germany. The synchrotron source employs the PETRA III U29 undulator with a gap of 20.95 mm, corresponding to an energy of 10 keV and a wavelength λ = 1.24 Å. Scattering intensities were recorded using a Pilatus 6M hybrid photon detector.

For PS20, measurements were performed at a sample-to-detector distance of 3 m, covering a *q* range of 0.03–4.43 nm^−1^, with a beam size at the sample of 250 × 100 µm. Due to the low sample viscosity, an autosampler was used and measurements were carried out in batch mode. For each sample, 40 µl was loaded into a flow-through quartz capillary and measured at 20°C, using an exposure time of 0.1 s per frame for 40 frames (total exposure of 4 s). For VitE-TPGS (0.4 wt%), samples were measured only with the laboratory SAXS instrument.

### 3.3. Data reduction

To extend the accessible *q* range, particularly at low scattering vector values in the SAXS regime, laboratory measurements collected at two different sample–detector distances (600 and 1800 mm) were merged.

To this end, the multiple acquisitions collected at each distance were first averaged to improve the signal-to-noise ratio. The averaging procedure was carried out using *PRIMUS* (*ATSAS*; Franke *et al.*, 2025 ▸; Manalastas-Cantos *et al.*, 2021 ▸), which performs a preliminary correlation-based comparison of all profiles to identify and exclude datasets affected by spurious signals or acquisition artifacts.

After averaging, the datasets acquired at 600 and 1800 mm were manually merged, with the scattering curves automatically rescaled by the *XSACT* software (https://www.xenocs.com/), so that they were quantitatively consistent across distances. A *q* range with a reasonably good signal-to-noise ratio in the low-*q* region – corresponding to the dataset collected at the longer distance – was selected and combined with the higher-*q* portion of the dataset collected at the shorter distance. This approach aims to produce the smoothest and most continuous profile possible across the full *q* range.

Raw SAXS detector frames collected at the synchrotron facility were reduced using the standard beamline pipeline based on *pyFAI* (https://github.com/silx-kit/pyFAI). 2D detector images were corrected for dark current, environment, detector geometry and masked pixels, and the *q* scale was calibrated using a silver behenate standard. Corrected 2D patterns were azimuthally integrated to obtain 1D intensity profiles 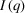 with polarization and solid-angle normalization. Intensities were normalized by incident beam flux and sample transmission, and solvent/background scattering measured under identical conditions was subtracted to isolate the sample signal. Data were placed on an absolute intensity scale using a calibrated reference standard (typically water). The final reduced 1D scattering curves were used for structural analysis.

### 3.4. Model-dependent fits of SAXS data

The detailed results obtained from the model-dependent fits of SAXS data have been reported in Section S4 of the supporting information. Here, we summarize the main findings.

**Figure 3:**
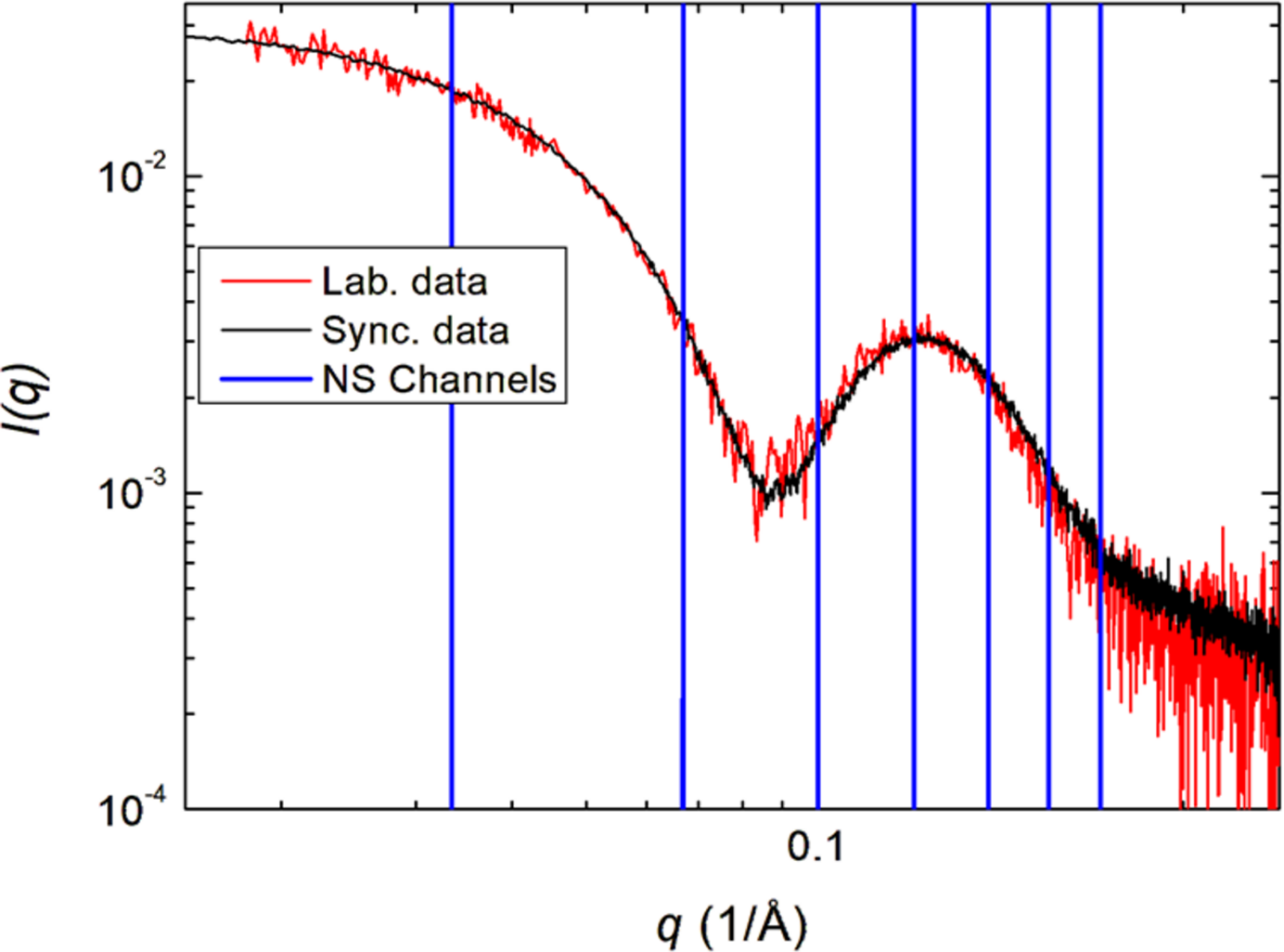
Experimental SAXS profiles for PS20 micelles (monomer concentration of 5 mg ml^−1^) measured in the laboratory (red) and at the synchrotron (black). The blue lines are the NS channels.

Fig. 3 ▸ compares the laboratory (red) and synchrotron (black) SAXS data for PS20 micelles (monomer concentration of 5 mg ml^−1^). The two datasets differ only in their noise level. The NS channels (blue lines), derived as previously described, are also shown. The results of the model-dependent fits reported in the supporting information, Section S4, show that also laboratory data are sufficiently robust to characterize the structure of core–shell spheroidal micelles. Despite the higher level of noise compared with synchrotron radiation, the laboratory measurements yield structural parameters consistent with those obtained at the synchrotron.

This is primarily due to the high oversampling ratio of the SAXS data, which provides enough mathematical redundancy to compensate for a lower signal-to-noise ratio. By applying appropriate smoothing techniques – leveraging the fact that the signal is sampled well beyond the Nyquist sampling rate – the underlying scattering profiles become nearly identical, allowing for a reliable retrieval of the morphology of nano-objects without significant loss of accuracy.

**Figure 4:**
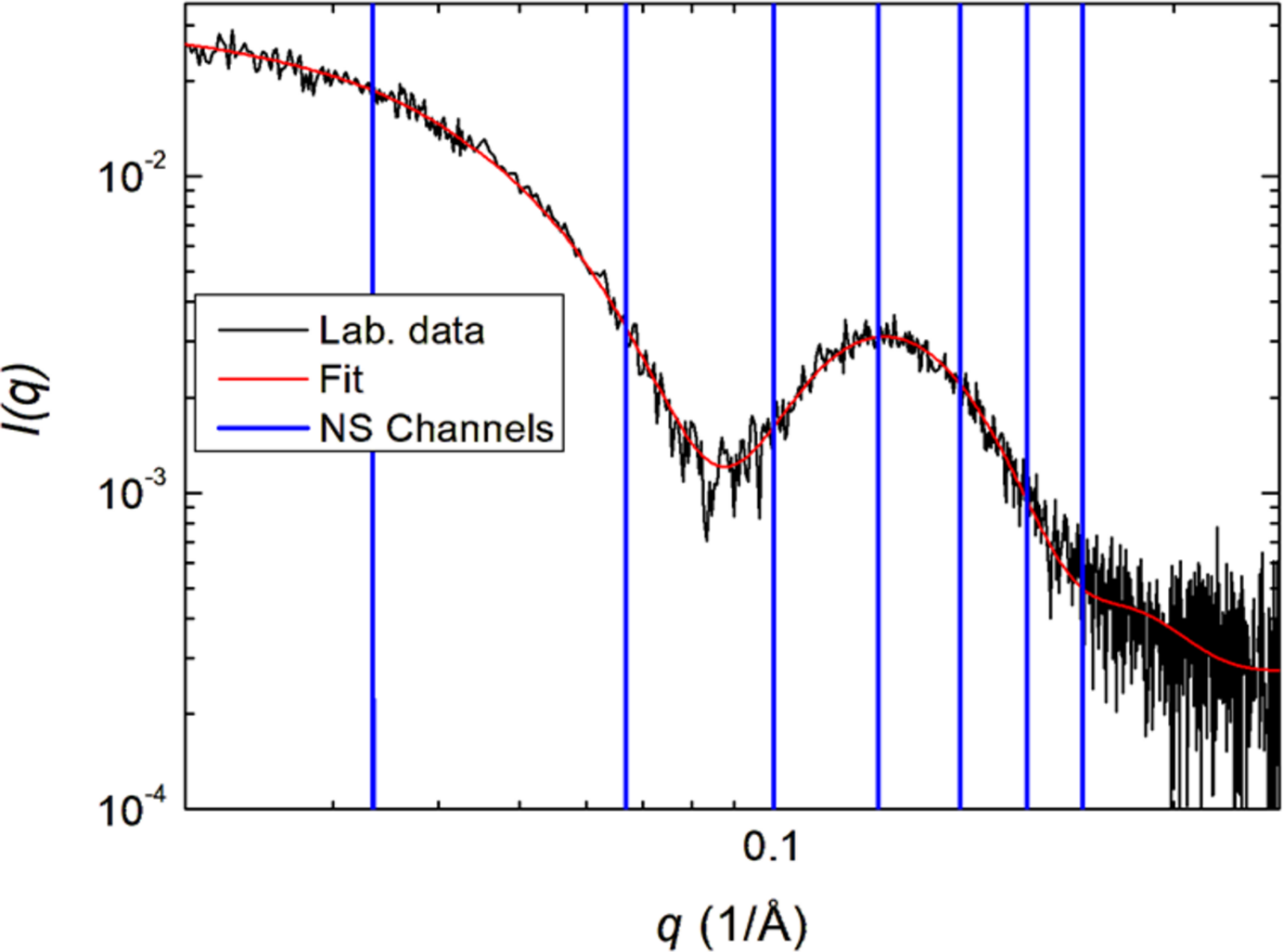
Experimental SAXS profiles for PS20 micelles (monomer concentration of 5 mg ml^−1^) measured in the laboratory (black) versus the profile calculated minimizing the χ^2^ (red), as described in detail in Section S4 of the supporting information. Vertical lines are the NS channels.

Fig. 4 ▸ shows the experimental laboratory SAXS data (black) compared with the fit (red) given by equations (S1)–(S21). Details of the fit have been reported in Section S4.

**Figure 5:**
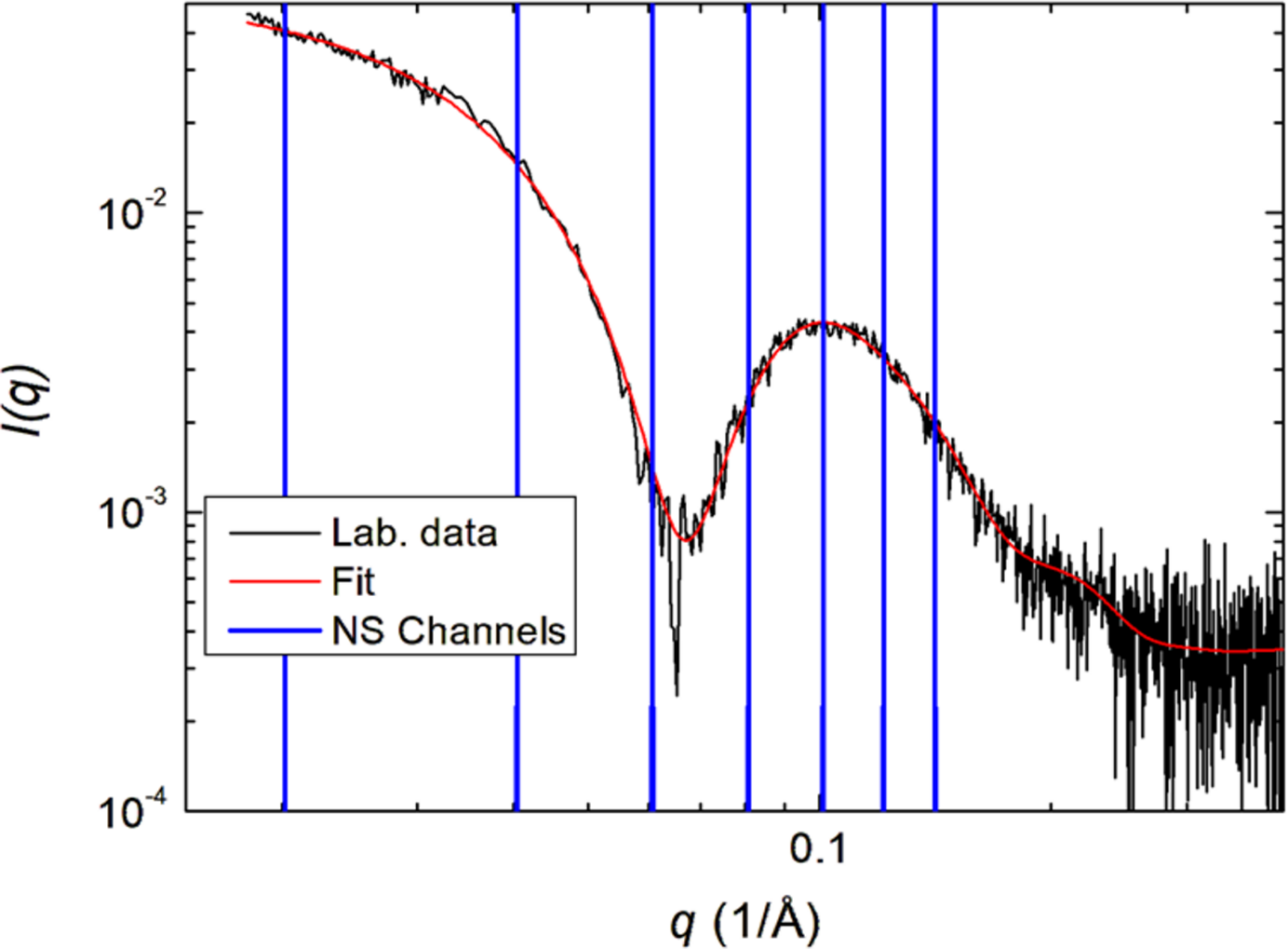
Experimental SAXS profiles for VitE-TPGS micelles measured in the laboratory (black) versus the profile obtained by the model-dependent fit (red), as described in detail in Section S4 of the supporting information. Vertical lines are the NS channels.

Fig. 5 ▸ shows the experimental laboratory SAXS data (black) compared with the model-dependent fit (red) obtained for VitE-TPGS micelles. Details of the fit have been reported in Section S4.

For a VitE-TPGS solution of the same concentration and for laboratory SAXS data, De Caro *et al.* (2025 ▸) found 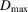 = 122.0 ± 0.1 Å, 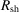
 = 16.7 ± 0.1 Å, *X* = 0.137 ± 0.002, ɛ = 1.51 ± 0.01, ɛ_M_ = 1.32 ± 0.02, *X*_ρ_ = −1.81 ± 0.05, *R*_g_ = 47.3 ± 0.5 Å and 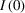 = 0.056 ± 0.001 cm^−1^, in agreement with the results obtained here from the laboratory data (see Tables 2–4 reported in the previous sections, and Table S3 of Section S4). Therefore, we can conclude that the SAXS profile determined in the laboratory contains all the scattering information needed for a fast and reliable structural solution of the micelles under study by means of the new proposed approach, under the assumptions of the simplified model discussed in the supporting information.

**Figure 6:**
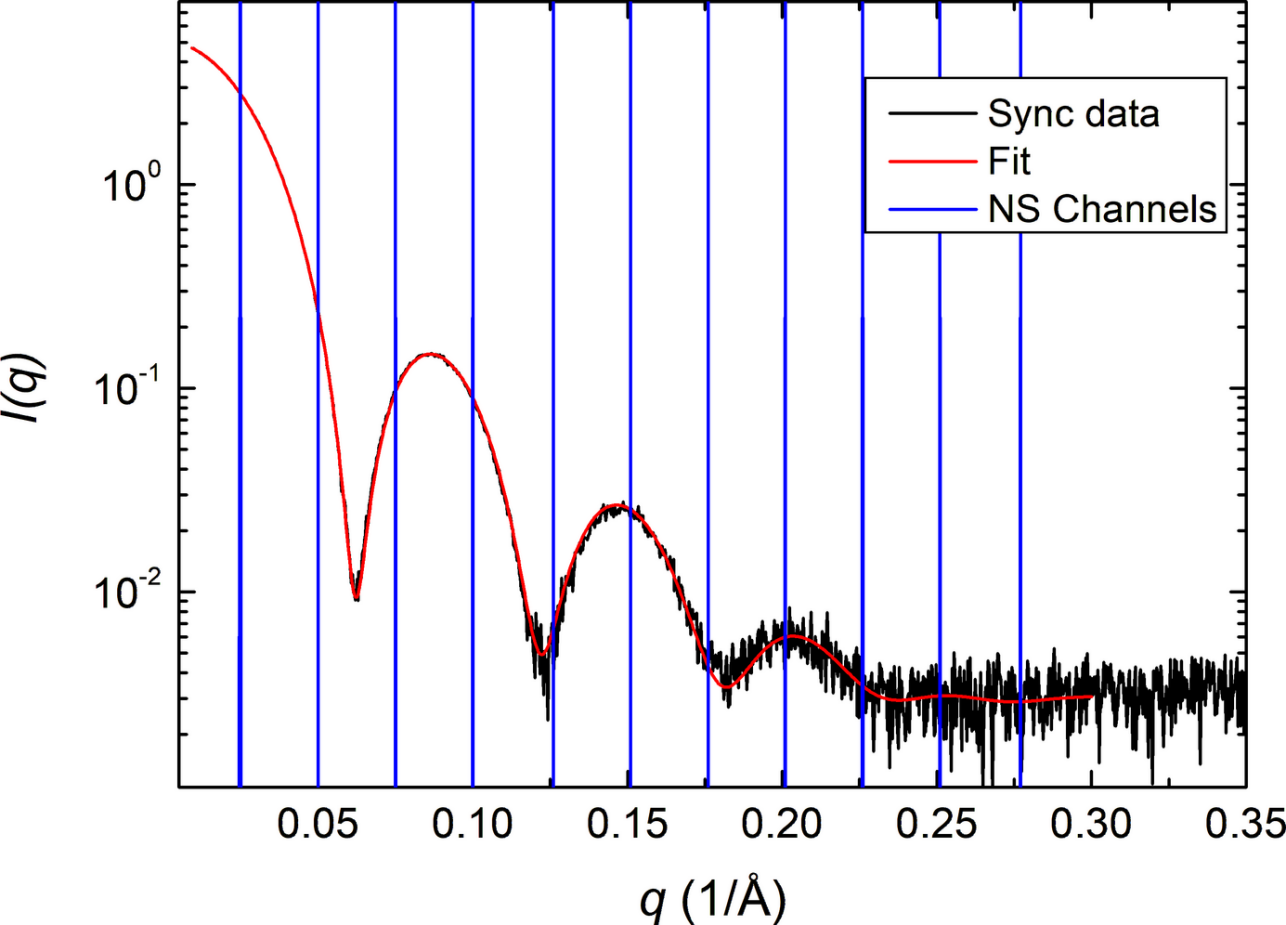
Experimental SAXS profiles for apoferritin (SASDFN8) (black) versus the profile (red) calculated by means of the model-dependent fit, described in Section S4 of the supporting information. Vertical lines are the NS channels.

Finally, in Fig. 6 ▸ we show the results of the model-dependent fit (red) for the SASDFN8 apoferritin SAXS data (black) derived from horse spleen, taken from the SASBDB repository. From Table 4 ▸, previously reported, and Table S3 of the supporting information, it is evident that there is overall good agreement between the results obtained using our approach and those reported in the SASBDB repository for the SASDFN8 apoferritin, particularly in the ‘.out file’ generated by *GNOM* (Svergun, 1992 ▸).

## 4. Related literature

The following references are cited in the supporting information: Doucet *et al.* (2025 ▸), Lipfert *et al.* (2007 ▸).

## 5. Conclusions

In this work, we have presented a method to reliably estimate 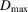 directly from the SAXS profile, using a new approach based on the Shannon sampling theorem. The proposed new approach enables the fast determination of several structural quantities characterizing the unknown structure of proteins as well as core–shell spheroidal micelles – such as mass, volume, ellipticity, maximum size, number of monomers, hydrated fraction and shell thickness – without the need to calculate the pair distribution function 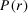. The application of the same formalism to relevant known proteins, taken from the SASBDB repository, has shown the reliability of the new approach in deriving the maximum size and mass of proteins. A new formula has been derived for determining the aggregation number of micelles starting from SAXS data on a relative scale. All these quantities can be derived from experimental SAXS data, through the Shannon sampling formalism. For SAXS data on an absolute scale also the shell and core electron-density difference can be derived without any model-dependent fit of SAXS data.

We have further demonstrated that the new proposed approach, even using laboratory SAXS data, can yield reliable morphological results, as validated using experimental measurements on PS20 and VitE-TPGS micelles, despite the higher noise level compared with synchrotron data. A good overall agreement has been obtained for the structural parameters derived for core–shell structures by the new approach both with the model-dependent fit of experimental SAXS data and with the results reported in the literature, under the assumptions of the simplified model discussed in the supporting information.

The approach discussed here, based on the Nyquist–Shannon sampling of the experimental SAXS profile, can be applied to nano-objects of any shape. The new proposed approach involves calculating integral quantities of the SAXS data, through the Nyquist–Shannon sampling formula. Consequently, it does not suffer from limiting effects due to the *q*-truncation of experimental data, as happens in the Fourier transform of SAXS data used for calculating the pair distance distribution function. Our tests have shown that the new proposed approach is able to give correct structural information about the scattering particles, without the need to calculate the pair distribution function or model-dependent fits of SAXS data. Therefore, this approach is quite useful for high-throughput data analysis, typical of massive SAXS collection (*in situ* and *operando* experiments). For core–shell micelles, the structural results obtained by the new approach could also be considered a useful preliminary and quantitative analysis of the structural features that could be refined/checked by a model-dependent fit of the experimental SAXS data.

More articulated modeling of micelle SAXS data, which could take into account complexity related to excluded volume and fluctuation effects in the corona (Svaneborg & Pedersen, 2000 ▸) or coupling of size dispersity and eccentricity (Lebecque *et al.*, 2017 ▸), could benefit from the preliminary quantitative results extracted by the Nyquist–Shannon-based analysis proposed here.

In particular, modeling of surfactant and polymeric micelles builds upon earlier work originally developed for the analysis of relatively noisy SANS data, and more recent approaches continue to evolve, improving information extraction even from sparse SANS datasets (Tung *et al.*, 2025 ▸). Nevertheless, a fast evaluation of some structural properties of micelles and proteins (maximum size, aggregation number of monomers, mass, volume), working directly on SAXS laboratory experimental data without any need of conversion of data in real space, is a useful tool. It can be combined with different techniques (such as SANS) when further characterization is required to define in more detail the structure of the scattering objects.

The new approach will be implemented in the software *SUNBIM* (Scattarella *et al.*, 2025 ▸). The future use of this software by users will help us to further understand the actual reliability of structural information obtained by this approach in (i) retrieving the structure of core–shell micelles without any model-dependent fitting of the SAXS data and (ii) determining useful preliminary structural information on proteins from SAXS data, such as molecular mass.

## Supplementary Material

Supporting information. DOI: 10.1107/S1600576726004358/fc5088sup1.pdf

## Figures and Tables

**Table 1:** 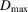
, *I*(0) and 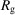 obtained by equations (3) and (5), compared with the values reported in the SASBDB repository for the proteins or obtained by model-dependent fits described in the supporting information for the core–shell nanostructures studied in this work The oversampling ratio and the estimation of the missing contributions – here defined as 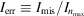 [equation (10)] and 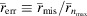 [equation (11)] – are reported in the first column. We have checked the reliability of the 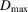 values obtained by equation (13) by determining it by model-dependent fits of SAXS data (values given in parentheses in the table), described in detail in the supporting information and in Section 3.

Structure code	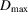 (Å) (GNOM†)	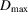 (Å) [equation (13), model-dependent fit from equations (S1)–(S21)]	*I*(*0*) (cm^−1^) (Guinier)	*I*(0) (cm^−1^) [equation (3)]	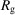 (Å) (Guinier)	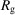 (Å) [equation (5)]
Proteins						
SASDUE4 (lysozyme): 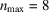 , OR = 14, 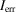 = −0.002, 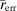 = −0.01	46.87 ± 0.44	46.7 ± 0.1	1.0070 ± 0.0006	1.050 ± 0.002	14.46 ± 0.013	14.34 ± 0.01
SASDFN8 (apoferritin): 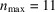 , OR = 90, 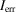 = −0.0005, 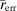 = −0.001	125.0 ± 0.14	126.4 ± 0.7 (126.0 ± 0.1)	5.070 ± 0.001‡	5.098 ± 0.005	52.37 ± 0.007	52.20 ± 0.007
SASDFP8 (carbonic anhydrase 2): 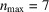 , OR = 222, 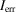 = −0.0045, 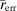 = −0.02	50.5 ± 0.2	50.9 ± 0.3	7744 ± 2	7788 ± 29	17.93 ± 0.008	18.03 ± 0.01
						
Micelles						
PS20 laboratory data: 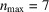 , OR = 63, 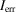 = −0.01, 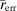 = −0.05	94.61 ± 0.56	95.1 ± 0.1 (94.5 ± 0.5)	0.0303 ± 0.0025	0.031 ± 0.002	35.91 ± 0.16	36.07 ± 0.38
PS20 synchrotron data: 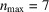 , OR = 115, 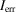 = −0.02, 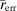 = −0.05	95.3 ± 0.45	94.7 ± 0.4 (96.0 ± 0.5)	0.03046 ± 0.0005	0.031 ± 0.001	35.59 ± 0.05	36.13 ± 0.01
VitE-TPGS laboratory data: 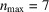 , OR = 48, 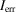 = −0.006, 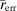 = −0.02	122.2 ± 0.14	120.9 ± 0.8 (121.5 ± 0.5)	0.0588 ± 0.0006	0.054 ± 0.003	47.91 ± 0.01	47.2 ± 0.4

† https://www.embl-hamburg.de/biosaxs/gnom_intro.html.

‡ The SAXS intensity for SASDFN8, reported in the SASBDB repository, has been multiplied by 0.0001 to express it in cm^−1^.

**Table 2:** 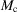
, 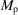 and 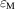 obtained by equations (22)–(26), compared with the mass *M* reported in the SASBDB repository, and the ellipticity 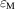 obtained by model-dependent fits (described in ssection S4 of the supporting information for the core–shell structures) *q*_max_ is given in units of Å^−1^.

Structure code	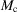 (kDa) [equations (22)–(24)]	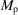 (kDa) [equation (25)]	*M* (kDa) (experimental value)	Mass density (g cm^−3^)	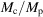 [ɛ_M_ for prolate core–shell structures, equation (26)]	ɛ_M_ (fit of SAXS data)
Proteins						
SASDUE4 (lysozyme): 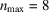 , 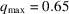	8.8 ± 0.1	14.2 ± 0.1	14.0	1.37	0.62 ± 0.01	
SASDFN8 (apoferritin): 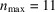 , 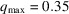	459.7 ± 2.0	452.3 ± 9.2	454	1.37	Core–shell^*a*^ 1.02 ± 0.06	1.02 ± 0.02
SASDFP8 (carbonic anhydrase 2): 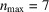 , 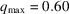	19.7 ± 0.4	29.4 ± 0.9	28	1.37	0.67 ± 0.02	
						
Micelles						
PS20 laboratory data: 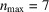 , 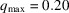	38.7 ± 4.8	26.7 ± 2.0	NA	1.15^*b*^	Core–shell 1.45 ± 0.09	1.39 ± 0.02
PS20 synchrotron data: 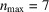 , 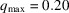	37.0 ± 0.1	25.0 ± 0.3	NA	1.15^*b*^	Core–shell 1.48 ± 0.01	1.45 ± 0.08
VitE-TPGS laboratory data: 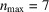 , 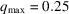	143.2 ± 14.8	102.6 ± 6.8	NA	1.08^*c*^	Core–shell 1.40 ± 0.06	1.36 ± 0.02

^*a*^Patriati *et al.* (2020 ▸), ^*b*^Le Maire *et al.* (2000 ▸), ^*c*^De Caro *et al.* (2025 ▸).

**Table 3:** Calculation of the volume and the number of monomers constituting the core–shell structures under study Equation (26) leads to two possible solutions. The actual shape, whether prolate or oblate, can be determined only through the estimation of the hydrated volume, which gives a physically reasonable value only for the correct shape. 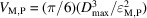, 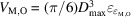, 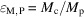, 
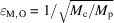
. *q*_max_ is given in units of Å^−1^, *V*_mon_ nm^3^, *M*_mon_ kDa, *c* mg ml^−1^.

Structure code	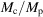	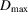 (Å) [equation (13)]	Prolate [equation (26)] 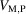 (nm ^3^)	Oblate [equation (26)] 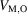 (nm ^3^)	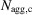 [equation (27)]	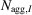 [equations (28)–(29)]	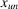 [equation (30)][Disp-formula fd30]: prolate, from 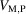 ; oblate, from 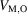
Proteins							
SASDFN8 (apoferritin): 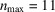 , 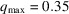 , *Z* ≃ 10^4^, *V*_mon_ ≃ 21.3, *M*_mon_ ≃ 20.0, *c* = 11	1.02 ±0.06	126.4 ± 0.7	1024 ± 38	1050 ± 19	No. of subunits 23.4 ± 0.7	No. of subunits 24.1 ± 0.2	Prolate 0.51 ± 0.01; oblate 0.53 ± 0.01
							
Micelles							
PS20† laboratory data: 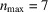 , 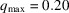 , *Z* = 670, *V*_mon_ ≃ 1.820, *M*_mon_ ≃ 1.2275, *c* = 5	1.45 ± 0.09	95.1 ± 0.1	215 ± 21	374 ± 9	46 ± 3	45 ± 1	Prolate 0.61 ± 0.04; oblate 0.78 ± 0.01
PS20† synchrotron data: 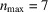 , 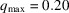 , *Z* = 670, *V*_mon_ ≃ 1.820, *M*_mon_ ≃ 1.2275, *c* = 5	1.48 ± 0.01	94.7 ± 0.4	253 ± 6	386 ± 5	45 ± 1	45 ± 1	Prolate 0.60 ± 0.01; oblate 0.78 ± 0.01
VitE-TPGS‡ laboratory data: 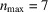 , 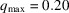 , *Z* = 844, *V*_mon_ ≃ 2.340, *M*_mon_ ≃ 1.544, *c* = 4	1.40 ± 0.06	120.9 ± 0.8	475 ± 39	784 ± 23	130 ± 8	127 ± 3	Prolate 0.36 ± 0.05; oblate 0.61 ± 0.01

† For the PS20 monomer we have assumed the following chemical formula: sorbitan monolaureate + 20 ethyl­ene oxide (EO) units: C_18_H_34_O_6_ + 20*x*(C_2_H_4_O) = C_58_H_114_O_26_.

‡ For the VitE-TPGS monomer we have assumed the following chemical formula: hydro­phobic chain + succinate linker + PEG 1000 (*n* = 23): C_29_H_50_O_2_ + C_4_H_4_O_3_ + C_46_H_92_O_23_ = C_79_H_146_O_28_.

**Table 4:** Core (ɛ) and micelle (ɛ_M_) ellipticity, 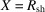
*/*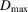 and shell thickness 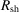 of core–shell structures derived by the new approach Comparison with the results obtained by the model-dependent simulations, described in the supporting information (Section S4), is also shown.

Structure code	ɛ_M_[equation (26)]	ɛ_M_ (fit of SAXS data)	ɛ [equation (S22)]	ɛ (fit of SAXS data)	*X* [solution of equation (31)]	*X* (fit of SAXS data)	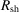 (Å) [solution of equation (31)]	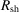 (Å) (fit of SAXS data)
Proteins								
SASDFN8 (apoferritin)	1.02 ± 0.06	1.02 ± 0.02	1.03 ± 0.02	1.03 ± 0.01	0.200 ± 0.005	0.184 ± 0.001	25.3 ± 0.6	23.2 ± 0.1
								
Micelles								
PS20 laboratory data	1.45 ± 0.09	1.39 ± 0.02	1.86 ± 0.14	1.75 ± 0.01	0.165 ± 0.005	0.170 ± 0.006	15.7 ± 0.3	16.1 ± 0.5
PS20 synchrotron data	1.48 ± 0.01	1.45 ± 0.08	1.91 ± 0.05	1.83 ± 0.01	0.160 ± 0.005	0.165 ± 0.005	15.2 ± 0.4	15.8 ± 0.5
VitE-TPGS	1.40 ± 0.06	1.36 ± 0.02	1.65 ± 0.07	1.63 ± 0.01	0.140 ± 0.005	0.155 ± 0.005	16.9 ± 0.5	18.8 ± 0.5

**Table 5:** Electron-density differences obtained for the core–shell structures 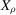
 has been obtained as the numerical solution of equation (32) and compared with the value obtained in the supporting information (section S4) by the model-dependent fits of SAXS data.

Structure code	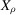 [solution of equation (32)]	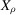 (fit of SAXS data)	Δρ_shell-solution_ (e Å^−3^) [equation (33)]	Δρ_core-solution_ (e Å^−3^) [equation (34)]
Proteins				
SASDFN8 (apoferritin)	−0.95 ± 0.01	−0.91 ± 0.01	0.090 ± 0.001	0.004 ± 0.002
				
Micelles				
PS20 laboratory data	−2.32 ± 0.01	−2.20 ± 0.05	0.021 ± 0.001	−0.027 ± 0.005
PS20 synchrotron data	−2.39 ± 0.01	−2.10 ± 0.05	0.023 ± 0.001	−0.031 ± 0.003
VitE-TPGS	−1.78 ± 0.01	−1.75 ± 0.05	0.030 ± 0.001	−0.023 ± 0.002

## Data Availability

Data are available from the SASBDB repository: https://www.sasbdb.org.
