# Supplementary material for: Shannon sampling based approach for the structural solution of nano-objects by laboratory and synchrotron SAXS data
Source: J Appl Crystallogr. 2026 Jul 7;59(Pt 4):1170–83. doi: 10.1107/S1600576726004358 (PMC13440053; doi:10.1107/S1600576726004358)
Supplement: Supplementary file 1 [file j-59-01170-sup1.pdf]

JOURNAL OF
APPLIED
CRYSTALLOGRAPHY

Volume 59 (2026)

Supporting information for article:

Shannon-sampling-based approach for the structural solution of nano-objects by laboratory and synchrotron SAXS data

Liberato De Caro, Domenico Alberga, Francesco Scattarella, Teresa Sibillano, Vincenzo Mangini, Boyang Zhou, Thibaud Stoll and Cinzia Giannini

The main aim of section S.1 is to provide all mathematical backgrounds for performing model-dependent simulation of SAXS data, used to fit SAXS experimental data and to have a further check of the results discussed in the main paper through the new proposed approach.

S1. Theoretical SAXS profile for ideal core-shell spheroidal nano-objects

The X-ray scattering amplitude of a particle with electron density ρ(r) is given by:

$$A(\boldsymbol{q}) = \int \left(\rho(\boldsymbol{r}) - \rho_s\right) exp(i\boldsymbol{q} \cdot \boldsymbol{r})\, d^3\boldsymbol{r}, \quad \text{(S1)}$$

and the SAXS intensity *I(q)* is the square of the scattered amplitude $A(\boldsymbol{q})$ averaged over all orientations. For a two-component spheroidal shape of semi-axes a_1 and c_1, an outer shell density ρ_1 and a core with semi-axes a and c and density ρ, the form factor is computed adding the amplitude contributions of each component (Lipfter et al. 2007):

$$I(q) = 3\int_0^1 dx \left(V\left(\rho - \rho_1\right)\frac{j_1(u)}{u} + V_1\left(\rho_1 - \rho_s\right)\frac{j_1(u_1)}{u_1}\right)^2, \quad \text{(S2)}$$

with j_1 the first-order spherical Bessel function, V=$4\pi a^2 c/3$ the volume of the core, and $V_1=4\pi a_1^2 c_1/3$ the volume of the whole micelle and

$$u = u\left(qR(x)\right) = q\left(a^2(1 - x^2) + c^2x^2\right)^{1/2}, \quad \text{(S3)}$$

$$u_1 = u\left(qR_1(x)\right) = q\left(a_1^2(1 - x^2) + c_1^2x^2\right)^{1/2}. \quad \text{(S4)}$$

The integral over x gives the average over all orientations.

With reference to Figure S1, let us define the electron-density differences through the following equations:

$$\Delta\rho_{core-shell} = \rho - \rho_1, \quad \text{(S5)}$$

$$\Delta\rho_{shell-solution} = \rho_1 - \rho_s. \quad \text{(S6)}$$

When we compare Eq. (S2) with the experimental SAXS data, a multiplicative constant is usually inserted as fitting parameter to minimize the χ^2(q) between experimental data and theoretical predictions. This implies that only the ratio of the two electron-density differences can be determined by minimizing the χ^2(q). For giving a value to the two electron-density differences a further constraint must be introduced, related to *I(0)*, if the SAXS measures are on a quantitative scale.

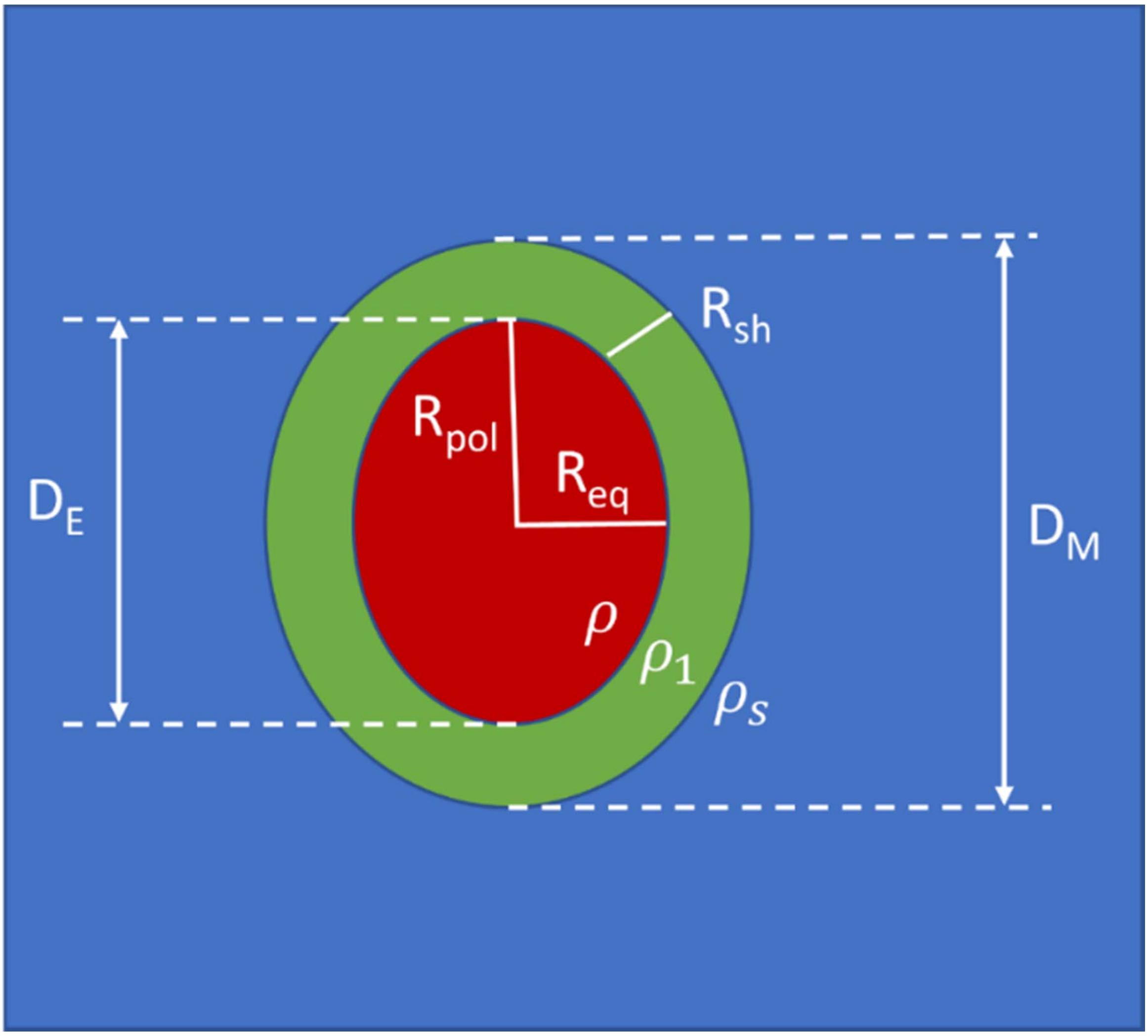

Figure S1 Core-shell prolate spheroidal micelle. The symbols D_M and D_E denote the micelle and core polar sizes, respectively. R_{pol} and R_{eq} are the polar and equatorial core radii, respectively.

Consequently, indicating with R_{sh} the shell thickness, D_M the micelle size along the polar axis, D_E the core size along the polar axis, ε the core ellipticity, ε_M the micelle ellipticity, D_{max} the maximum micelle size, we can put:

$$c/a = \varepsilon, \tag{S7}$$

$$2c = D_E = D_M - 2R_{sh} = (1 - 2X_\varepsilon)D_M, \tag{S8}$$

$$2a = D_E/\varepsilon, \tag{S9}$$

$$X = R_{sh}/D_{max}, \tag{S10}$$

$$X_\varepsilon = X/\min(1.0, \varepsilon_M), \tag{S11}$$

$$D_M = \min(1.0, \varepsilon_M)D_{max}, \tag{S12}$$

$$2c_1 = D_M, \tag{S13}$$

$$2a_1 = \frac{D_M}{\varepsilon}\left(1 - 2X_\varepsilon(1 - \varepsilon)\right), \tag{S14}$$

$$\varepsilon_M = c_1/a_1 = \frac{\varepsilon}{1+2(\varepsilon-1)X_\varepsilon} \ , \tag{S15}$$

$$X_\rho = \Delta\rho_{core-shell}/\Delta\rho_{shell-solution} \ . \tag{S16}$$

It should be noted that if the shell has a constant size along the polar and equatorial axes, the micelle ellipticity is different from the core ellipticity. From the above formula it follows that for prolate spheroidal shapes ($\varepsilon > 1$) the micelle ellipticity is

$$\varepsilon_M = \frac{\varepsilon}{1+2(\varepsilon-1)X}. \tag{S17}$$

For oblate spheroidal shapes ($\varepsilon < 1$)

$$\varepsilon_M = \varepsilon + 2(\varepsilon - 1)X. \tag{S18}$$

We note that, for constant shell sizes, the micelle ellipticity ε_M is always smaller than the core ellipticity ε.

Thus, by using the above expressions, Eq. (S2-S3) can be rewritten as follows:

$$I(q) = const \times \int_0^1 dx \left(\varepsilon \left(\frac{1-2X_\varepsilon}{\varepsilon} \right)^3 X_\rho \frac{j_1(u)}{u} + \left(\frac{1-2X_\varepsilon}{\varepsilon} + 2X_\varepsilon \right)^2 \frac{j_1(u_1)}{u_1} \right)^2, \tag{S19}$$

$$u = q \frac{D_{max}}{2} \left(\left(\frac{1-2X_\varepsilon}{\varepsilon} \right)^2 (1-x^2) + (1-2X_\varepsilon)^2 x^2 \right)^{1/2}, \tag{S20}$$

$$u_1 = q \frac{D_{max}}{2} \left(\left(\frac{1-2X_\varepsilon}{\varepsilon} + 2X_\varepsilon \right)^2 (1-x^2) + x^2 \right)^{1/2}. \tag{S21}$$

The above theoretical SAXS intensity depends on the following dimensionless quantities: ε, X_ε, X_{EP}, and on the maximum micelle size D_{max}. In the main paper we have shown how to estimate D_{max} directly by the experimental measured SAXS profile without calculating the $P(r)$.

We have derived the relationship between the ellipticity of the whole core-shell structure and the corresponding value for the core, both for prolate (Eq. (S17)) and the oblate (Eq. (S18)) shapes, assuming the shell thickness constant. These two equations can be inverted, giving the core ellipticity ε as a function of the ellipticity ε_M and of the ratio $X = R_{sh}/D_{max}$ of the shell thickness over the maximum size.

For prolate shapes we obtain:

$$\varepsilon = \frac{\varepsilon_M \times (1-2X)}{1-2X\varepsilon_M}. \tag{S22}$$

For oblate shapes we obtain:

$$\varepsilon = \frac{\varepsilon_M - 2X}{1-2X}. \tag{S23}$$

The core volume for the two shapes can be readily derived.

For prolate shapes we obtain:

$$V_{core} = \frac{\pi}{6} \times [(1-2X) \times D_{max}]^3 \times \frac{1}{\varepsilon^2}, \tag{S24}$$

with ε given by Eq. (S22).

For oblate shapes we obtain:

$$V_{core} = \frac{\pi}{6} \times [(1-2X) \times D_{max}]^3 \times \varepsilon, \tag{S25}$$

with ε given by Eq. (S23).

The hydrated volume of the core-shell structure can be calculated by Eq. (30) of the main paper

$$V_{hyd}=x_{hyd} \times V_M, \tag{S26}$$

and the shell volume is given by

$$V_{sh}=V_M - V_{core}, \tag{S27}$$

where V_M is the micelle volume. The volume $V_{mon,sh}$ of the part of the monomer constituting the core-shell structures, which is in the shell, can be estimated from the ratio of core volume and the non-hydrated fraction of the shell volume:

$$V_{mon,sh} = V_{mon} \times \frac{V_{core}}{(1-x_{hyd})\times V_{sh}}\,. \tag{S28}$$

The above equation gives the possibility to derive the fraction X by imposing

$$\Delta V=V_{sh} - V_{hyd} - N_{agg,I} \times V_{mon,sh} = 0. \tag{S29}$$

Eq. (S29) can be solved numerically, determining X and, consequently, the shell thickness R_{sh}, through the definition $X = R_{sh}/D_{max}$ as shown in the main paper

S2. Calculated SAXS profiles for non-ideal core-shell spheroidal nano-objects: a q-adaptive non-linear filtering approach

Given the SAXS theoretical profile $I(q)$, calculated for an ideal structure of core-shell spheroidal nano-objects, we should consider core and/or shell polydispersity (PD), or other structural inhomogeneities (e.g. smooth interface/surfaces) to obtain χ^2 values close to 1 (i.e., for obtaining the structural solution), where:

$$\chi^2 = \frac{1}{N_q-1-n_{fit}}\sum_{i=1}^{N_q}\left(\frac{I_{exp}(q_i)-I(q_i)}{\sigma_i}\right). \tag{S30}$$

Here, $I_{exp}(q_i)$ is the experimental SAXS intensity at the scattering vector q_i, σ_i is the experimental error on the measured intensity, N_q is the total number of experimental data; n_{fit} is the number of parameters used in the fit, the index i is running on all the measured data. $I(q_i)$ are obtained by Eqs. (A19-A21).

All these inhomogeneity effects cause a smoothing of structural SAXS fringes; to consider this smearing effect, several approaches have been already developed and implemented in software now available, such as SASView (Doucet et al. 2025). In this work we modelled not the cause but the effect of these structural imperfections, to calculate the smoothed SAXS profiles $I(q_i)_f$.

The smoothed SAXS profile $I(q_i)_f$ is obtained through a q-adaptive filter, determining the more effective filter shape minimizing the χ^2, according to the following equation

$$I(q)_f = (1 - w(\bar{q}))I(q) + w(\bar{q})\langle I(q)\rangle_m + I_{bk} \,, \tag{S31}$$

where:

$$w(\bar{q}) = \frac{1}{1+\left(\frac{1-\bar{q}}{\bar{q}}\times\frac{\bar{q}_0}{1-\bar{q}_0}\right)^{\alpha}} \,. \tag{S32}$$

The adjective "q-adaptive" means that not all the intensities are filtered at the same extent, but the actual weight of the filter in modifying the theoretical SAXS profile depends on the *q* value and increases at larger q. Here, $\bar{\boldsymbol{q}} = \boldsymbol{q}/\boldsymbol{q_{max}}$, where q_{max} is the maximum q-scattering vector used in calculation of χ^2. I_{bk} is a background intensity (fitting parameter). The exponent α and $\bar{\boldsymbol{q}}_\mathbf{0}$ define the level of nonlinearity of the filter $\boldsymbol{w}(\bar{\boldsymbol{q}}) = \frac{1}{1+\left(\frac{1-\bar{q}}{\bar{q}}\times\frac{\bar{q}_0}{1-\bar{q}_0}\right)^{\alpha}}$. For α=1 and $\bar{\boldsymbol{q}}_\mathbf{0} = \mathbf{1/2}$ we obtain a linear filter. For α=1 and $\bar{\boldsymbol{q}}_\mathbf{0} \neq \mathbf{1/2}$ we obtain a slightly non-linear filter. For α>>1 we have a sigmoidal filter centered at $\bar{\boldsymbol{q}}_\mathbf{0}$. $\langle I(q)\rangle_m$ is the mean-filter value of $I(q)$ over a range of *m* points (range of *q* values). Also, *m* and $\bar{\boldsymbol{q}}_\mathbf{0}$ are fitting parameters. Instead, in the sigmoid we can put α=1/*X*, i.e., the inverse of the shell-size divided by the micelle maximum size. The α parameter influences the sharpness of the sigmoidal filtering function. The relation α=1/*X* allows the proposed *q*-adaptative sigmoidal filter should be particularly suitable for describing smoothing effects due to the shell PD. Indeed, the filter is working only when $\boldsymbol{q} \geq \bar{\boldsymbol{q}}_\mathbf{0}$. In turn, this implies that not all the distances are affected by the PD. This usually happens when the core has a definite shape and size, and it is the shell thickness affected by variations. A wider application of the q-adaptive non-linear filtering approach to more experimental cases could be useful to clarify eventual relationships between these fitting parameters and PD values, but this finding goes beyond the scope of the present work.

In the case α=1 the whole q-range is affected by the non-ideal-structure effects, although intensities at high q-values would be more affected than low q-values. In turn, this could be related to the fact that all the distances are affected by PD, how it usually happens when the core is not well-defined in shape and size, but it is affected by PD. Since α and $\bar{\boldsymbol{q}}_\mathbf{0}$ are fitting parameters, this approach allows us to directly determine what kind of filtering is more suitable for the nano-objects under study. In any case, we could also allow the parameter α to vary from 1 to 1/*X*, or also to larger values, if needed.

As we will show in the following section, the comparison with the SASView fits indicated that the linear adaptive filtering α=1 has been more suitable when the SAXS profiles have been obtained on structures characterized by core PD. Conversely, the non-linear sigmoidal filtering,

with α=1/*X*, has been more suitable when the SAXS profiles have been obtained on structures characterized by shell-size PD. In any case, the versatility of the proposed q-adaptive filtering approach allows us to modify the calculated SAXS profile only in the q-ranges where the effects of the non-ideality of the nano-objects alters the experimental SAXS profiles, leaving unchanged the calculated intensities in other q-ranges where the differences between theoretical and experimental profiles are less evident.

The same approach can be used for a q-range selective smoothing of the SAXS experimental profiles, for a reliable derivation of the maximum distance and a statistically robust derivation of the Shannon channels' intensities, which are needed for predicting several fundamental structural properties of proteins and spheroidal core-shell micelles through SAXS profiles, without any need of performing model-dependent simulations, as discussed in the main paper.

S3. Check the correct scale of SAXS intensity errors

The SAXS laboratory data used in this study have been obtained combining two different measures, taken at two different distances: 600 and 1800 mm. Given this combined SAXS experimental profile we have the possibility to estimate errors on the measured intensities by comparing them with a denoised version of SAXS measured profile, obtained by applying a mean-filter of suitable width *m* (number of averaged points) on the experimental profile:

$$I(q)_{denoised} = \langle I(q)_{exper} \rangle_m. \quad \text{(S33)}$$

Given the smoothed profile (Eq. A31), the estimated intensity errors can be calculated as follows:

$$\Delta I(q)_{denoised} = \left| I(q) - \langle I(q)_{exper} \rangle_m \right|. \quad \text{(S34)}$$

One should use the maximum *m* value that does not cause the presence of structures in $\Delta I(q)_{denoised}$, related to the intensity maxima. In other words, the structure of $\Delta I(q)_{denoised}$ must be as flat as possible as a function of q. Our tests on the experimental data used in this study have shown that a suitable range of points over which to calculate mean values through the mean-filter for our laboratory data is m~30. For synchrotron data we have found m~20, values well below the oversampling ratio defined in the main paper.

We can compare the errors so obtained with those given by the instruments that have measured the SAXS profiles: $\Delta I(q)_{exp}$. If these experimental errors are correctly evaluated, we should have that the condition:

$$\Delta I(q)_{denoised}/\Delta I(q)_{exp} \leq 3 \quad \text{(S35)}$$

should be violated very rarely. Indeed, if we assume a normal distribution of the measured intensities with respect to the mean values, one will have a probability of only 0.27% to violate the above inequality

(3 standard deviation more than the mean value). Therefore only 0.27 % of the total number of experimental data N_q, from a statistical point of view, can violate the above inequality. We can rescale the $\Delta I(q)_{exp}$ values until the above condition is satisfied, multiplying the errors by a suitable Scale Factor (SF), thus obtaining the correctly rescaled errors:

$$\sigma(q) = err(q)_{correct} = SF \times \Delta I(q)_{exp} , \qquad \text{(S36)}$$

There the $\sigma(q)'s$ are used in the χ^2 calculations (Eq. (S30)). If SF=1 the $\Delta I(q)_{exp}$ have been correctly estimated. If SF>1 they are underestimated. If SF<1 they are overestimated. For the PS20 and VitE-TPGs laboratory data, obtained combining two different measures, at 600 and 1800 mm, we have found SF=1.02 and SF=1.0, respectively, thus confirming that the merging procedure has been correctly performed. For PS20 synchrotron data we have found SF=1.0. For the apoferritin synchrotron data we have found SF=1.15.

S4. Solution preparation and determination of the structural parameters of the core-shell structures

S4.1. PS20 micelles

PS20 was prepared as a 5 mg/mL aqueous solution. A 50 mg/mL stock solution was first prepared by dissolving 0.5 g of PS20 (CAS Nr 9005-64-5) in 10 g of ultrapure water under magnetic stirring at 400 rpm for 10 min at room temperature. The stock solution was subsequently diluted to the working concentration by mixing 500 µL of stock solution with 4.5 mL of ultrapure water, yielding a final concentration of 5 mg/mL. Prior to SAXS measurements, the solution was filtered through Axiva sterile cellulose acetate syringe filters with a pore size of 200 nm to remove any particulate matter.

Table S1 shows the results obtained on the PS20 micelles by the model-dependent fits. To assess the reliability of the data-merging procedure we have applied the constraint given by Eq. (S35).

Table S1 Summary of the micelle shape parameters obtained by PS20 laboratory and synchrotron data. PD=polydispersity. CD=Combined data measured at two Distances: 600 and 1800 mm.

Data	ε core	ε_M Micelle<br>Eq. (S17)	D_{max}(Å)<br>Fit<br>Eq. (S1-S21)	D_{max}(Å)<br>From $P(r)$<br>GNOM	R_{sh}(Å)<br>X	X_ρ
Laboratory CD<br>Eqs. (S1-S21)<br>χ^2 =1.05	1.75±0.01	1.39±0.02	94.5±0.5	94.61±0.56	16.06±0.5<br>0.170±0.006	-2.20±0.05
Laboratory CD<br>SASView Fit (*):<br>χ^2 =1.28;<br>PD=15% on the core equat. size;<br>D_{max}=94.0 ±2.0 Å	1.86±0.04	1.45±0.08	/	/	15.92±0.52<br>0.165±0.010	-2.22±0.22
Synchrotron	1.83±0.01	1.44±0.02	96.0±0.5	95.30±0.5	15.84±0.5	-2.10±0.05

Eqs. (A1-A21)<br>$\chi^2 = 1.20$	0.165±0.005

(*) (Doucet et al. 2025).

Moreover, to check the validity of results obtained by the laboratory measurements, we have repeated the same analysis on synchrotron SAXS data collected on PS20 micelles (prepared at the same concentration of 5 mg/mL). We have applied the fitting formulae given by Eq. (S1-S21) to compute the SAXS profile and compare the results with those obtained by the SASView program. Finally, as additional check, we have used the software GNOM (https://www.embl-hamburg.de/biosaxs/gnom_intro.html) for calculating independently structural quantities such as D_{max}, R_g and $I(0)$, to be compared with those calculated by our approach, in particular by Eq. (5) for the gyration radius, by Eq. (9) for the nano-object maximum size D_{max} *a*nd by Eq. (3) for $I(0)$.

The minimum of the chi-square (Eq. A30) has been obtained for the q-adaptive almost linear filter: α=1 in Eq. (S32). Even if we have the intensities corresponding to only 7 Shannon channels in the measured SAXS profile, Table 6 shows an overall good agreement between all the performed tests, and the reliability of Eq. (3), (5) and (9) in evaluating $I(0)$, r_g and D_{max}, respectively, directly from the measured SAXS profile, without any need to calculate the $P(r)$.

Figure 3 of the main paper shows the comparison of the laboratory (red) and synchrotron (black) SAXS data for PS20 micelles (monomer concentration of 5 mg/mL). The only distinction between the two measurements lies in their noise level. Also, the Shannon channels (blue lines) are shown.

The results reported in Table S1 show that also laboratory data are reliable for determining the structure of core-shell spheroidal micelles, even if the level of noise is higher than that of the synchrotron measures. The possibility of applying the Shannon sampling theorem to smoothed SAXS data allows us to reduce the effect of higher levels of noise and to obtain reliable structural solutions of the nanostructures under investigation even by laboratory SAXS data.

Figure 4 of the main paper shows the experimental laboratory SAXS data (black) compared to the fit (red) given by Eqs. (S1-S21). To obtain the estimated intensities used in Eq. (3) and (5) of the main paper, up to the maximum Shannon channel for which a SAXS signal was measured, a suitable averaging window, of approximately $m \approx 20$ points, was used to compute the mean-filter for the laboratory data. For synchrotron data we have m~15. In both cases these values are well below the oversampling ratio defined in the main paper. For the laboratory SAXS data we have found that the chi-square minimum is reached if we apply an almost-linear q-adaptive filter (α=1), with an offset of $q_0 = 0.254$ Å^{-1} , where $q_0 = \bar{q}_0 * q_{max}$. This implies that pair distances smaller than $2\pi/q_0$ =24.6 Å are those mainly affected by the filtering. For the synchrotron SAXS data we have found that the chi-square minimum is reached always if we apply an almost-linear q-adaptive filter (α=1), but with an offset of

$q_0 = 0.115$ Å^{-1} . This implies that pair distances smaller than $2\pi/q_0$ =54.6 Å are those mainly affected by the filtering.

S4.2. VitE-TPGS (0.4 wt%) micelles

VitE-TPGS was prepared in a 50 mM KH_2PO_4 buffer at pH 6.8. The buffer solution was obtained by dissolving 1360 mg of potassium dihydrogen phosphate and 180 mg of sodium hydroxide pellets in 200 mL of Milli-Q water, and stirring until the pH stabilized at 6.8, corresponding to a final phosphate concentration of 22.5 mM. For the sample, 400 mg of VitE-TPGS was added to 100 g of the prepared buffer and stirred for at least 40 min to ensure complete dissolution. The final pH was verified, and the solution was stored at 4°C prior to measurements. For filtration, an aliquot of the sample was passed four times through Axiva sterile cellulose acetate syringe filters with a pore size of 200 nm.

Table S2 reassumes the results obtained on the VitE-TPGS micelles. Figure 5 of the main paper shows the experimental laboratory SAXS data (black) compared to the fit (red) given by Eqs. (S1-S21).

Table S2 Summary of the micelle shape parameters obtained by VitE-TPGS laboratory data. PD=polydispersity. CD=Combined data measured at two distances: 600 and 1800 mm.

Data	ε core	ε_M Micelle Eq. (S17)	D_{max}(Å) Fit Eq. (S1-S21)	D_{max}(Å) From $P(r)$ GNOM	R_{sh}(Å) / X	X_ρ
Laboratory CD Eq. (S1-S21) χ^2 =1.20 Eq. (S1-S21)	1.63±0.01	1.36±0.02	121.5±0.5	122.2±0.8	18.8±0.5 / 0.155±0.005	-1.75±0.05
Laboratory CD SASView Fit (*): χ^2 =1.42; PD=40% on the shell size; D_{max}=115.4±4.6	1.8±0.1	1.49±0.02	"	"	15.07±0.7 / 0.131±0.011	-1.94±0.07

(*) (Doucet et al. 2025).

It should be noted that the SAXS profile determined in laboratory contains all scattering information needed to retrieve the correct structural solution of the micelles under study, as discussed in the main paper. For obtaining the estimated intensities used in Eq. (3) and (5) of the main paper, up to the 7th Shannon channel, a mean-filter with *m*~20 has been used. SASView gives a larger χ^2 value, and a very large shell PD. In our model-dependent fit, we have obtained that a *q*-adaptive sigmoidal filter minimizes the χ^2, with an offset of $q_0 = 0.123$ Å^{-1}. This implies that only pair distances smaller than $2\pi/q_0$ =51.2 Å are affected by the filtering.

S4.3. Apoferritin

We tested our approach also on the SASDFN8 nanostructure, an apoferritin from horse spleen, taken from the SASBDB repository (https://www.sasbdb.org/data/SASDFN8/). Table S3 reassumes the obtained results, and Figure 6 of the main paper shows the experimental SAXS data and the obtained model-dependent fit. The results reassumed in Table S3 have been obtained minimizing the χ^2, applying an almost linear q-adaptive filter (α=1) (Eq. A1-A21): $\bar{q}_0 = 0.55, q_0 = 0.165$ Å^{-1}.

Table S3 Summary of the apoferritin shape parameters obtained by synchrotron data.

Data	ε core	ε_M Micelle Eq. (S17)	D_{max}(Å) Fit Eq. (S1-S21)	D_{max}(Å) From $P(r)$ SASBDB	R_{sh}(Å)<br>X	X_ρ
Synchrotron Eq. (S1-S21) χ^2 =1.10	1.03±0.01	1.02±0.02	126.0±0.1	125.0±0.5	23.2±0.1<br>0.184±0.001	-0.91±0.01

The results show that pair distances smaller than $2\pi/q_0$ =38.0 Å are those mainly affected by the filtering procedure. With this respect, let us note that in the case of the apoferritin the actual structure is not a sphere, as assumed in the simplified SAXS modeling here used, since the structure is composed of 24 subunits that assemble with 432 (octahedral) symmetry. Approximating the actual structure with a sphere leads to a "fictitious" variation of the nano-object size, whose effect onto the SAXS curve could be assimilated to a non-ideality of the sphere. Between the most protruding points (toward the 3-fold symmetry vertices) and the most recessed ones (along the 4-fold pores), the overall thickness difference is approximately 10 Å. i.e., causing a variation of pair-distances of about 20 Å over the whole nano-structure size. The effects of this variation of the actual apoferritin nanostructure on the SAXS profile, with respect to the approximating core-shell spherical structure in the SAXS modeling here used, has required the use of a suitable filtering of the calculated ideal $I(q)$.

As previously discussed (Eq. (S34-S36), we have the possibility to estimate errors on the measured intensities $\Delta I(q)_{exp}$ by comparing them with the difference of the measured $I(q)$ with a denoised version of it, lets say $I(q)_{smooth}$, obtained by applying a mean-filter of suitable width on the experimental profile. Thus, we can rescale the $\Delta I(q)_{exp}$ values by a suitable Scale Factor (SF) until the errors on the measured intensities are in scale with the differences between $I(q)$ and $I(q)_{smooth}$. We obtained χ^2 =1.10, for SF=1.15 applied to errors on the measured intensity (Eq. (A34-A36)). Without applying any rescaling of the intensity errors, i.e. for SF=1.0, we would have χ^2 =1.45. Considering the approximated model – a core-shell sphere - with which we have modelled the actual apoferritin nanostructure also a χ^2 =1.45 could be considered satisfactory.

From Table S3, it is evident that there is overall good agreement between the results obtained using our approach and those reported in https://www.sasbdb.org/data/SASDFN8/, particularly in the ".out file" generated by GNOM (Svergun 1992). It should be noted that apoferritin exhibits a nearly spherical shape, and the value $X_{\rho} \sim -1$ indicates a slightly positive core electron-density difference — that is, an electron density slightly higher than that of the solvent. Our analysis also reproduces the spherical shape of apoferritin, as determined at the SAXS resolution, without imposing this constraint, since the measured ellipticity was $\varepsilon_M = 1.02 \pm 0.02$.

Figure S2 shows the Kratky plot (Glatter and Kratky, 1982) for SASDFN8 apoferritin.

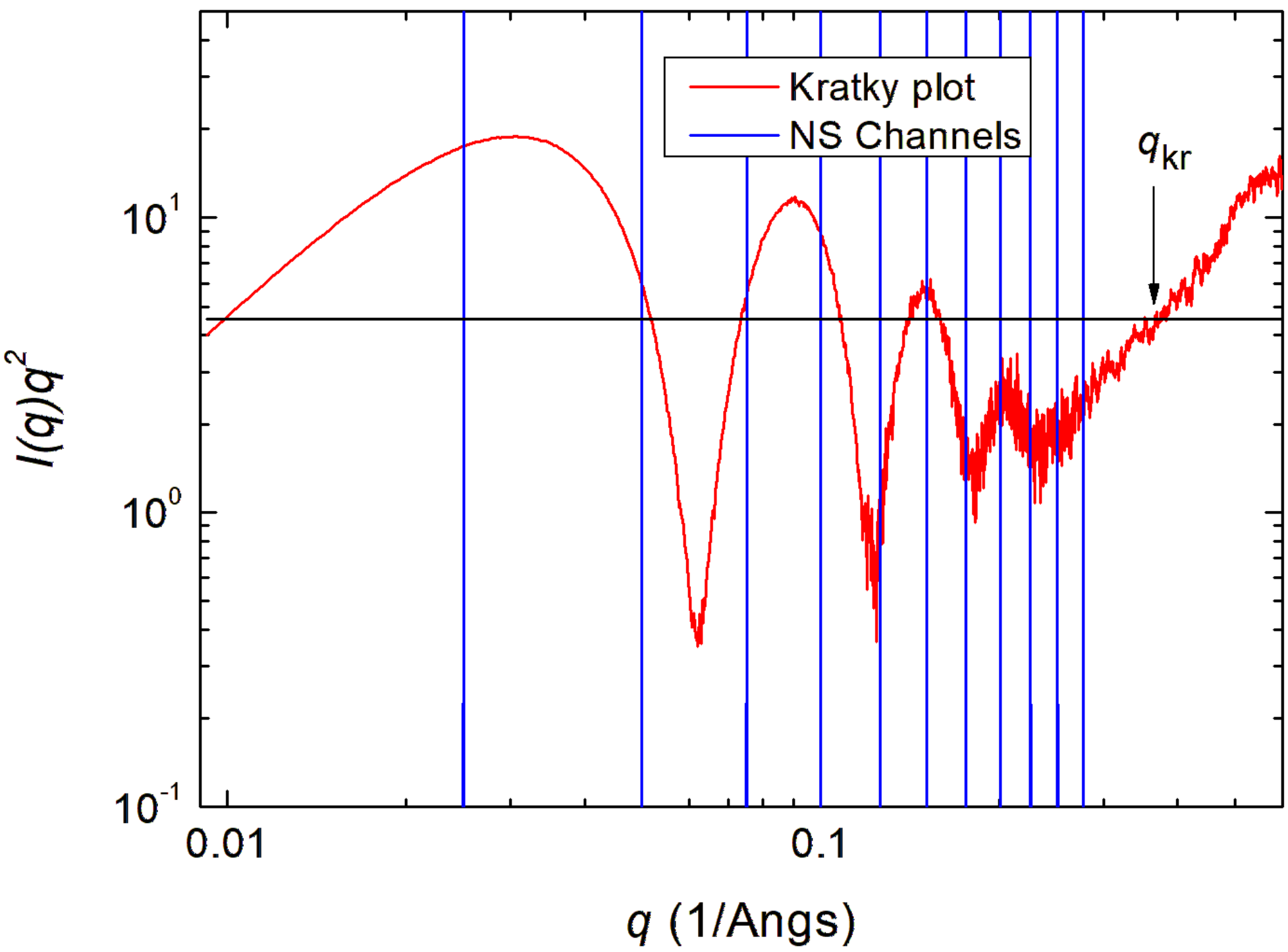

Figure S2 Kratky plot for apoferritin. The black line shows the mean value $\langle I(q)q^2 \rangle$. q_{kr} is the scattering vector where the level of noise prevents to have further structural information from the $I(q)q^2$ profile.

The black line shows the mean value $\langle I(q)q^2 \rangle$, used for determining the maximum scattering vector q_{kr} used for the calculation of the Porod invariant Q, as explained in the main paper. q_{kr} is an estimate of the scattering vector beyond which the level of noise prevents of having further structural information from the $I(q)q^2$ profile.

S5. Derivation of the polar/equatorial asymmetry of core-shell nano-objects from the ratio of mass M_c/M_p

From Eq. (21) and (22) of the main paper we have

$$M_c = \frac{1}{123.1} \times \frac{V^2}{4\pi^2 l_c^2 R_g}. \quad \text{(S37)}$$

Moreover, l_c can be expressed also as ratio of the two invariants (Grant 2022):

$$l_c = \pi \times \frac{\int_0^\infty I(q) q dq}{\int_0^\infty I(q) q^2 dq}. \quad \text{(S38)}$$

The above equation can be numerically integrated, after having calculated the form factor of a homogeneous spheroid by performing an orientational average of the theoretical squared amplitude, in analogy of Eq. (S19-S21). The two integrals in Eq. (S38), expressed in terms of sums on Shannon channels can be integrated over a grid up to a $n_{\max}$=200, already sufficiently high for the convergence.

Furthermore, for a spheroidal shape it can be shown that (De Caro et al. 2025)

$$R_g = \sqrt{\frac{2+\varepsilon^2}{20}}\,. \quad \text{(S39)}$$

Table S4 shows l_c, R_g and $l_c^2 R_g$ for a spheroidal shape with ellipticity ε_M, i.e. with a polar/equatorial axes' ratio ε_M.

Within the numerical approximations used for deriving the calculated quantities, the last column of Table S4 shows that:

$$\frac{[l_c^2 R_g]_{spheroid}}{[l_c^2 R_g]_{sphere}} \cong \varepsilon_M. \quad \text{(S40)}$$

Moreover, for a prolate spheroid we have:

$$V_{Prolate} = \frac{\pi}{6}\frac{D_{max}^3}{\varepsilon_M^2}, \quad \text{(S41)}$$

i.e., its volume scale as $1/\varepsilon_M^2$ with respect to volume of a sphere with the same maximum size.

For an oblate spheroid we have:

$$V_{Oblate} = \frac{\pi}{6} D_{max}^3 \varepsilon_M. \quad \text{(S42)}$$

i.e., its volume scales as ε_M with respect to volume of a sphere with the same maximum size.

Table S4 l_c , R_g and $l_c^2 R_g$ for a spheroidal shape. The last column shows $l_c^2 R_g$ divided by the corresponding value for a sphere and normalized to the ellipticity ε_M, i.e., to the polar/equatorial axes' ratio.

ε	l_c/a	R_g/a	$(\frac{[l_c^2 R_g]_{spheroid}}{[l_c^2 R_g]_{sphere}})/\varepsilon_M$
0.5	0.574	0.335	1.005
0.6	0.622	0.344	1.007
0.7	0.662	0.353	1.004
0.8	0.696	0.363	0.999
0.9	0.727	0.375	1.000
1.0	0.750	0.387	1.001
1.1	0.777	0.401	0.999
1.2	0.798	0.415	1.000
1.3	0.817	0.430	1.002
1.4	0.834	0.445	1.005
1.5	0.850	0.461	1.009
1.6	0.864	0.477	1.013
1.7	0.877	0.494	1.017
1.8	0.889	0.512	1.021
1.9	0.900	0.530	1.026
2.0	0.910	0.548	1.031

Therefore, for a prolate shape we have:

$$\left(\frac{[l_c^2 R_g]_{Prolate}}{[l_c^2 R_g]_{sphere}}\right)^{-1} \times \left(\frac{V_{Prolate}}{V_{sphere}}\right)^{-1} \cong \frac{1}{\varepsilon_M} \times \varepsilon_M^2 = \varepsilon_M. \quad \text{(A43)}$$

For an oblate shape we have:

$$\left(\frac{[l_c^2 R_g]_{spheroid}}{[l_c^2 R_g]_{sphere}}\right)^{-1} \times \left(\frac{V_{spheroid}}{V_{sphere}}\right)^{-1} \cong \frac{1}{\varepsilon_M} \times \frac{1}{\varepsilon_M} = \frac{1}{\varepsilon_M^2}. \quad \text{(A44)}$$

M_c can be related to $\left(\frac{[l_c^2 R_g]_{spheroid}}{[l_c^2 R_g]_{sphere}}\right)^{-1}$. Since the effective volume related to the Porod invariant is that of an equivalent sphere with the same scattering of the particle, M_p can be related to $\frac{V_{spheroid}}{V_{sphere}}$. Therefore, we have shown that the ratio of M_c and M_p for spheroidal core-shell nano-objects should be related just to the polar/equatorial asymmetry of the shape, as verified in the experimental tests discussed in the main paper:

$$M_c/M_p = \begin{cases} \varepsilon_M & \varepsilon_M \geq 1 \\ 1/\varepsilon_M^2 & \varepsilon_M < 1 \end{cases}. \quad \text{(S45)}$$

S6. Derivation of the core and shell electron-density differences for core-shell nano-objects when SAXS data on an absolute scale are available

For a core-shell structure of spheroidal shape the theoretical expression of the squared gyration ratio is given by (De Caro et al. 2024):

$$R_{g,th}^2 = \frac{(2+\varepsilon^2)(D_{max}-2R_{sh})^5 X_\rho + D_{max}(D_{max}+2(\varepsilon-1)R_{sh})^2[\varepsilon^2 D_{max}^2 + 2(D_{max}+2(\varepsilon-1)R_{sh})^2]}{20\varepsilon^2[(D_{max}-2R_{sh})^3 X_\rho + D_{max}(D_{max}+2(\varepsilon-1)R_{sh})^2]} \quad \text{(S46)}$$

with ε given by Eq. (S22) for a prolate shape and Eq. (S23) for an oblate one, and X_ρ - the ratio of the two electron-density differences - given by Eq. (s16). After having determined the maximum size, the shell thickness, the ellipticity of the core-shell structure under study, we can compare the theoretical value of the gyration ratio given by Eq. (S46) with the normalized gyration ratio determined by the Shannon-sampling approach of the SAXS data

$$\bar{r}_{g,th}^2 = \frac{R_{g,th}^2}{D_{max}^2} = \frac{\sum_{n=1}^{n_{max}}\left(1-\frac{6}{(n\pi)^2}\right)(-1)^{n+1}I_n}{2\sum_{n=1}^{n_{max}}(-1)^{n+1}I_n}. \qquad \text{(S47)}$$

The above expression can be considered as an equation that can be numerically solved for determining X_ρ, as discussed in the main paper.

This ratio can be determined also on SAXS data on a non-absolute scale. However, to determine the shell- and core-electron-density differences we need SAXS data on an absolute scale.

If we have available absolute-scale SAXS data, from Eq. (A3) of (De Caro et al. 2024) we can derive the following equations:

$$\Delta\rho_{shell-solution} = \frac{1}{V_M + X_\rho \times V_{core}}\sqrt{\frac{M_{mon} N_{agg,c} I(0)}{c N_A {r_e}^2}}, \qquad \text{(S48)}$$

$$\Delta\rho_{core-solution} = \left(1 + X_\rho\right) \times \Delta\rho_{shell-solution}. \qquad \text{(S49)}$$

Here, c is the monomer concentration (g/cm^3), $N_A = 6.02214 \cdot 10^{23}$/mol is the Avogadro number, r_e is the classical electron radius ($2.81794 \cdot 10^{-13}$ cm/e), M_{mon} is the molar mass of one monomer, and $N_{agg,c}$ is the number of aggregated monomers within the core-shell structure, given by Eq. (27) of the main paper; V_M is the micelle volume and V_{core} is the core volume.

References

De Caro, L., Stoll, T., Grandeury, A., Gozzo, F., & Giannini, C. (2024). Characterization of Surfactant Spheroidal Micelle Structure for Pharmaceutical Applications: A Novel Analytical Framework. Pharmaceutics, 16, 604. https://doi.org/10.3390/pharmaceutics16050604

De Caro, L., Stoll, T., Grandeury, A., Gozzo, F., & Giannini, C. (2025). Characterization of VitE-TPGS Micelles Linked to Poorly Soluble Pharmaceutical Compounds Exploiting Pair Distribution Function's Moments. Pharmaceutics, 17, 431. https://doi.org/10.3390/pharmaceutics17040431

Doucet, M., Adams, M., Agouzal, N., Alina, G., Anuchitanukul, A., Attala, Z., Bakker, J., Beaucage, P., Berger, J., Bourne, R., Bouwman, W., Bressler, I., Butler, P., Cadwallader-Jones, I., Campbell, K., Cho, J.-H., Cooper-Benun, T., Cortes Hernandez, R., Doutch, J., … Zheng, A. (2025). SasView version 6.0.1 (6.0.1). https://doi.org/10.5281/zenodo.14940319

Glatter, O., & Kratky, O. (1982). Small-Angle X-ray Scattering. Academic Press.

Grant, T. D. (2022). Describing small-angle scattering profiles by a limited set of intensities, J. Appl. Cryst., 55, 1116–1124. https://doi.org/10.1107/S1600576722006598

Lipfert, J., Columbus, L., Chu, V. B., Lesley, S. A., & Doniach, S. (2007). Size and Shape of Detergent Micelles Determined by Small-Angle X-ray Scattering, J. Phys. Chem. B, 111, 12427-12438. 10.1021/jp073016l

Svergun, D. I. (1992). Determination of the regularization parameter in indirect-transform methods using perceptual criteria. Journal of Applied Crystallography, 25, 495–503. https://doi.org/10.1107/S0021889892001663
